# A Comprehensive Review on Pharmacotherapeutics of Herbal Bioenhancers

**DOI:** 10.1100/2012/637953

**Published:** 2012-09-17

**Authors:** Ghanshyam B. Dudhatra, Shailesh K. Mody, Madhavi M. Awale, Hitesh B. Patel, Chirag M. Modi, Avinash Kumar, Divyesh R. Kamani, Bhavesh N. Chauhan

**Affiliations:** Department of Pharmacology & Toxicology, College of Veterinary Science & Animal Husbandry, Sardarkrushinagar Dantiwada Agricultural University, Sardarkrushinagar 385506, Gujarat, India

## Abstract

In India, Ayurveda has made a major contribution to the drug discovery process with new means of identifying active compounds. Recent advancement in bioavailability enhancement of drugs by compounds of herbal origin has produced a revolutionary shift in the way of therapeutics. Thus, bibliographic investigation was carried out by analyzing classical text books and peer-reviewed papers, consulting worldwide-accepted scientific databases from last 30 years. Herbal bioenhancers have been shown to enhance bioavailability and bioefficacy of different classes of drugs, such as antibiotics, antituberculosis, antiviral, antifungal, and anticancerous drugs at low doses. They have also improved oral absorption of nutraceuticals like vitamins, minerals, amino acids, and certain herbal compounds. Their mechanism of action is mainly through absorption process, drug metabolism, and action on drug target. This paper clearly indicates that scientific researchers and pharmaceutical industries have to give emphasis on experimental studies to find out novel active principles from such a vast array of unexploited plants having a role as a bioavailability and bioefficacy enhancer. Also, the mechanisms of action by which bioenhancer compounds exert bioenhancing effects remain to be explored.

## 1. Introduction

Modern pharmaceutical research is concerned with all aspects of identifying new chemical substances with new modes of action. Particularly, economics of treatment linked to drug dosage has led to new drug development technologies. As a result, treatments are now becoming more affordable for wide sections of society, including the financially challenged. One way to achieve reduction in drug dosage, and therefore drug toxicity and cost, is to increase drug bioavailability [1]. The consumption of antibiotics and drugs by man is increasing at an alarming rate. Out of the total drugs and chemicals, 20–50% of that use is unnecessary depending on the class of antibiotic. In addition, indiscriminate use of antibiotics promotes antibiotic resistance leading to multiple drug resistance and makes it difficult to control the diseases. The infected individuals have to consume more amount of antibiotics; this may be due to (1) reduced absorption in gut membrane when taken orally, (2) restrictive uptake by target microbe, and (3) operation of efflux pump leading to indiscriminate extrusion of the antibiotics or therapeutic molecules. So, the major amount of the applied drug is wasted and only a minor amount is being targeted to the site of infection [2].

In addition, the unutilized drug/antibiotics amount remains as a load in the body and environment acting as a selection pressure facilitating emergence of drug resistance, ultimately leading to failure of antibiotics against resistant infections. This is also responsible for side effects, illness, and reduction in life expectancy. One of the possible ways to reduce drug dosage is synergism between two therapeutic agents, that is, combination therapy. However, if both drugs used concurrently have antimicrobial property, the problem of selection pressure and drug toxicity will continue. Thus, there is need of molecules, which are not antimicrobial or target drugs but enhance activity and availability of main drugs in combination therapy, that is, bioenhancers. These molecules by their presence will not exert any selection pressure for mutants to emerge resistant against them and on that their ill effects can be minimized. The resistance development process will be substantially delayed, ultimately leading to enhanced life-span of the novel and existing antibiotics. Such drugs/molecule facilitators should have novel properties such as (1) nontoxic to humans or animals, (2) they should be effective at a very low concentration in a combination, (3) they should be easy to formulate, and (4) most importantly enhance uptake/absorption and activity of the drug molecules. This can lead to developing judicious and strategic concentrations of antibiotic with specific bioenhancers to improve availability of the drug for controlling the infectious organisms effectively [2].

Herbal bioenhancer is an agent of herbal origin or any phytomolecule, which is capable of enhancing bioavailability and bioefficacy of a particular drug or nutrient with which it is combined, without any typical pharmacological activity of its own at the dose used. In the 1920's, Bose, an acknowledged author of “Pharmacographia Indica,” reported an enhanced antiasthmatic effect of an Ayurvedic formula containing *vasaka* (*Adhatoda vasica*) when administered with long pepper [3]. The term bioavailability enhancer was first coined by Indian Scientists at the Regional Research laboratory, Jammu (RRL, now known as Indian Institute of Integrative Medicine) discovered and scientifically validated piperine as the world's first bioavailability enhancer in 1979 [4].

The concept of bioenhancers of herbal origin can be tracked back from the ancient knowledge of Ayurveda system of medicine. Use of ayurvedic preparation “Trikatu” from the period between the 7th century B.C. and the 6th century A.D., which is a Sanskrit, word meaning three acrids. It refers to a combination of black pepper (*Piper nigrum *Linn.), long pepper (*Piper longum *Linn.), and ginger (*Zingiber officinale *Rosc.), which contains active component piperine, which enhances the bioavailability of drugs, nutrients, and vitamins [2].

## 2. Bioavailability/Bioefficacy-Enhancing Activity

The term bioavailability or bioenhancing activity is defined as “a substance at a lower dosage level, which in combination with a drug or nutrient provides more availability of the drug by reducing the consumption of the drug or nutrient resulting in enhanced efficacy of the drugs.”

The great interests for the improvement of bioavailability of a large number of drugs are (1) poorly available, (2) administered for long periods, (3) toxic, and (4) expensive. Maximizing bioavailability is therapeutically important because the extent of bioavailability directly influences plasma concentrations and consequently therapeutic efficacy. Bioavailability enhancement can make the expensive drugs affordable and reduce the toxic effects by reducing the required dose of drugs.

Poorly bioavailable drugs remain subtherapeutic because a major portion of a dose never reaches the plasma or exerts its pharmacological effect unless and until very large doses are given which may lead to serious side effects. Any significant improvement in bioavailability will result in lowering the dose or the dose frequency of that particular drug. Intersubject variability is particularly of concern for a drug with a narrow safety margin. Incomplete oral bioavailability includes poor dissolution or low aqueous solubility, poor intestinal membrane permeation, degradation of the drug in gastric or intestinal fluids, and presystemic intestinal or hepatic metabolism. Many therapeutic treatments are also accompanied by loss of essential nutraceuticals in the course of therapy. The bioenhancers improve nutritional status by increasing bioavailability/bioefficacy of various nutraceuticals including metals and vitamins [5].

Bioavailability enhancement can be done by the following [5].

- Promoting the absorption of the drugs from GIT.
- Inhibiting or reducing the rate of biotransformation of drugs in the liver or intestines.
- Modifying the immune system in such a way that the overall requirement of the drug is reduced substantially.
- Increasing the penetration or the entry into the pathogens even where they become persistors within the macrophages such as for *Mycobacterium tuberculosis *and such others. This eventually ensures the enhanced killing of these organisms is well secured within the places otherwise inaccessible to the active drug.
- Inhibiting the capability of pathogens or abnormal tissue to reject the drug, for example, efflux mechanisms frequently encountered with antimalarial, anticancer and antimicrobial drugs.
- Modifying the signaling process between host and pathogen ensuring increased accessibility of the drugs to the pathogens.
- Enhancing the binding of the drug with the target sites such as receptors, proteins, DNA, RNA, and the like in the pathogen, thus potentiating and prolonging its effect leading to enhanced antibiotic activity against pathogens.
- Besides above mode of action, the bioenhancer agents may also be useful for promoting the transport of nutrients and the drugs across the blood brain barrier, which could be of immense help in the control of diseases like cerebral infections, epilepsy, and other CNS problems.

Modern drug development processes achieve oral bioavailability enhancement by a number of approaches.

- Increasing the polarity of the drug through chemical modification.
- Salt preparation or complexation.
- Prodrug formation.
- Micronization and nanonization.
- Specific polymorphic form selection.
- Targeted delivery of the drug to the site of action.
- Controlled drug delivery through film coating.
- Sustained drug release through polymorphic matrices formation.
- Liposomal microencapsulation and so forth.
- Application of P-glycoprotein inhibitors [6, 7].

However, bioavailability enhancement through the supplementation of the main therapeutic agent with a secondary agent gained wide popularity since the traditional times. However, based on Ayurvedic literature, a new approach is increasing bioavailability of drugs including poorly bioavailable drugs by using herbal bioenhancers [5].

Major categories of drugs that have shown increased bioenhancement include cardiovascular, respiratory, CNS, GIT, antibiotics, and anticancerous. Some examples include tetracyclines, sulfadiazine, vasicine, rifampicin, pyrazinamide, ethambutol, phenytoin, phenobarbitone, carbamazepine, nimesulide, indomethacin *β*-carotene, coenzyme *Q*_10_, ciprofloxacin, curcumin, dapsone, amino acids, glucose, and several other classes of drugs [8].

## 3. Mechanisms of Action

Herbal bioenhancers act through several mechanisms of action. Different herbal bioenhancers may have same or different mechanisms of action. They increase bioavailability of nutraceuticals by acting on gastrointestinal tract to enhance absorption, whereas they increase bioavailability of drugs by acting on drug metabolism process. Various mechanisms of action postulated for herbal bioenhancers are shown in Table 1.

They cause inhibition of gastric emptying (GE) of solids/liquids in rats and gastrointestinal transit (GT) in mice in a dose- and time-dependent manners was studied. It significantly inhibited GE of solids and GT at the doses extrapolated from humans (1 mg/kg and 1.3 mg/kg *p.o.* in rats and mice, resp.). However, at the same dose the effect was insignificant for GE of liquids. One-week oral treatment of 1 mg/kg and 1.3 mg/kg in rats and mice, respectively, did not produce a significant change in activity as compared to single-dose administration. GE inhibitory activity is independent of gastric acid and pepsin secretion [14].

Thermogenic and bioenergetic mechanisms are believed to be triggered by activation of thermoreceptors and release of catecholamines and/or direct action as beta 1, 2, 3-adrenoceptor agonist. Secretion of catecholamines can also be mediated by ATP via P_2_-type purinergic receptors and through a direct or indirect stimulation by the compositions of the invention of dopaminergic and serotinergic systems. It is known that stimulation of *β*-3 adrenoceptors results in increased thermogenesis, decrease in the amount of white adipose tissue without food intake being affected, increased levels of insulin receptors, and decreased levels of serum insulin and blood glucose. This may possess antiobesity and antidiabetic effects, which by themselves contribute to the mechanism of thermogenesis and the increase in lean body mass. The thermogenic effect may also be mediated by an increase in the activity of thyroid peroxidase, an important enzyme in thyroid hormone synthesis, an increase in the plasma levels of triiodothyronine (T_3_) and thyroxine (T_4_) with simultaneous increase in tissue oxygen uptake, and increase in thermogenesis [19].

Piperine mediated changes in the permeability of rat intestinal epithelial cells: the status of *γ*-glutamyl transpeptidase activity, uptake of amino acids, and lipid peroxidation was studied. The effect of piperine on the absorptive function of the intestine were studied. *In vitro* experiments showed that piperine (25–100 *μ*M) significantly stimulated *γ*-glutamyl transpeptidase activity, enhanced the uptake of radiolabelled l-leucine, l-isoleucine, and l-valine, and increased lipid peroxidation in freshly isolated epithelial cells of rat jejunum. In the presence of benzyl alcohol, an enhanced *γ*-glutamyl transpeptidase activity due to piperine was maintained. These results suggested that piperine may interact with the lipid environment to produce effects which lead to increased permeability of the intestinal cells [12].

## 4. Herbal Bioenhancers

### 4.1. Piperine

Piperine, the major plant alkaloid present in *P. nigrum *Linn (Black pepper) and *P. longum *Linn (Long pepper), has bioavailability enhancing activity for some nutritional substances and for some drugs [20]. It has been used extensively as a condiment and flavoring for all types of savory dishes. Piper species have been used in folklore medicine for the treatment of various diseases, including seizure disorders [21]. Piperine is known to exhibit a variety of biological activities which include anti-inflammatory activity [22, 23], antipyretic activity [24], fertility enhancement [25], antifungal activity [26], antidiarrhoeal activity [27], antioxidant activity [28–33], antimetastatic activity [34], antithyroid activity [35, 36], antimutagenic activity [37–40], antitumor activity [39, 41, 42], antidepressant activity [43–45], antiplatelet activity [46], analgesic activity [47], hepatoprotective activity [48], antihypertensive activity [49], and antiasthmatic activity [50]. Piperine exhibits a toxic effect against hepatocytes [51] and cultured hippocampal neurons [52], reproductive toxicity in swiss albino mice [53], and immunotoxicity [54].

Piperine reduces the aflatoxin B_1_-induced cytotoxicity and micronuclei formation in rat hepatoma cells in concentration-dependent manner. It is capable of counteracting aflatoxin B_1_ toxicity by suppressing cytochromes P450-mediated bioactivation of the mycotoxin [55]. Inhibition of aflatoxin B_1_-induced cytotoxicity and genotoxicity in chinese hamster cells by piperine was reported by Reen et al. [56]. A significant suppression (33.9–66.5%) in the micronuclei formation induced by benzo(a)pyrene and cyclophosphamide was reduced following oral administration of piperine at doses of 25, 50, and 75 mg/kg in mice [32].

Piperine modulates the oxidative changes by inhibiting lipid peroxidation and mediating enhanced synthesis or transport of glutathione thereby replenishing thiol redox [57]. Simultaneous supplementation with black pepper or piperine in rats fed high fat diet lowered thiobarbituric acid reactive substances (TBARS) and conjugated dienes levels and maintained superoxide dismutase (SOD), catalase (CAT), glutathione peroxidase (GPX), glutathione-S-transferase (GST), and glutathione (GSH) levels close to controls in rats [30]. Piperine could effectively inhibit benzo(a)pyrene-induced lung carcinogenesis in albino mice by offering protection from protein damage and also by suppressing cell proliferation [58]. It produces chemopreventive effect by modulating lipid peroxidation and augmenting antioxidant defense system [59, 60]. Supplementation of piperine causes inhibition of Phase I and II enzymes, elevation of glutathione metabolizing enzymes, reduction in DNA damage, and DNA protein cross-links in benzo(a)pyrene-induced lung carcinogenesis in mice [38, 61].

The antiapoptotic efficacy of piperine has been demonstrated against cisplatin-induced apoptosis via heme oxygenase-1 induction in auditory cells [62]. Piperine can reverse the corticosterone induced reduction of brain-derived neurotrophic factor mRNA expression in cultured hippocampal neurons [44]. Gallic acid exerts a synergistic effect when administered with piperine and provides a more pronounced therapeutic potential in reducing beryllium-induced hepatorenal dysfunction and oxidative stress consequences [63]. Piperine contains pentacyclic oxindole group which is effective for immunomodulation. This immunomodulation activity is due to its multifaceted activities such as antioxidative, antiapoptotic, and restorative ability against cell proliferative mitogenic response, thymic and splenic cell population, and cytokine release [64].

Daily supplement taken with a nutrient or nutrients by an average healthy adult, piperine is effective and safe in a broad dose range. A preferred effective dose range of piperine for oral use to enhance gastrointestinal nutrient absorption is 0.0004–0.15 mg/kg/day. The recommended dose of piperine for a healthy individual for oral use is approximately 5 mg/person/day. Black pepper contains approximately 5–9% piperine, listed by the Food and Drug Administration (FDA) as an herb which is generally recognized as safe (GRAS) for its intended use as spice, seasoning, or flavoring. The bioenhancing dose of piperine is approximately 15 mg/person/day and no more than 20 mg/day in divided doses, which corresponds to from several thousands to up to 40,000 times less than the LD_50_ dose of piperine, as established in various experiments on rodents. The effective bioenhancing dose of piperine for drug compounds varies, but the prior art studies indicated that a dose of approximately 10% (w/w) of the active drug could be regarded as an appropriate bioenhancing dose for most drugs [19]. LD_50_ of piperine has been found to be 330 and 514 mg/kg in mice and rats, respectively. In subacute toxicity tests, piperine in a dosage of 100 mg/kg was found to be nontoxic [65].

There are two possible explanations for the role of piperine in drug bioavailability: (a) nonspecific mechanisms mainly promoting rapid absorption of drugs and nutrients, for example, increased blood supply to the gastrointestinal tract, decreased hydrochloric acid secretion which prevents breakdown of some drugs, increased emulsifying content of the gut, and increased enzymes like *γ*-glutamyl transpeptidase which participate in active and passive transport of nutrients to the intestinal cells, and (b) nonspecific mechanisms inhibiting enzymes participating in biotransformation of drugs, preventing their inactivation and elimination [19].

#### 4.1.1. Drug Metabolizing Enzymes Inhibited by Piperine (Shown in Table 2)

The interaction of piperine with enzymatic drug biotransforming reactions in hepatic tissue has been studied *in vitro* and *in vivo*. Piperine inhibited arylhydrocarbon hydroxylation, ethylmorphine-N-demethylation, 7-ethoxycoumarin-O-deethylation, and 3-hydroxy-benzo(a)pyrene glucuronidation in rat postmitochondrial supernatant *in vitro* in a dose-dependent manner. Piperine inhibition of these reactions in postmitochondrial supernatant from 3-methylcholanthrene and phenobarbital treated rats was similar to the controls. Inhibition by piperine of arylhydrocarbon hydroxylase (AHH) from 3-methylcholanthrene treated rats was comparable to that observed with 7, 8-benzoflavone. Piperine caused noncompetitive inhibition of hepatic microsomal AHH from the untreated and 3-methylcholanthrene-treated rats with a *K*_*i*_ = 30 *μ*M which was close to the apparent *K*_*m*_ of AHH observed in the controls. Similarly, the kinetics of inhibition of ethylmorphine-N-demethylase from control rat liver microsomes exhibited noncompetitive inhibition with an apparent *K*_*m*_ = 0.8 mM and *K*_*i*_ = 35 *μ*M. These studies demonstrated that piperine is a nonspecific inhibitor of drug metabolism. Oral administration of piperine in rats strongly inhibited the hepatic AHH and Uridine diphosphate-(UDP-) glucuronyltransferase (UGT) activities. The maximal inhibition of AHH observed within 1 h restored to normal value in 6 h. These results demonstrate that piperine is a potent inhibitor of drug metabolism [15]. Piperine modified the rate of glucuronidation by lowering the endogenous UDP-glucuronic acid (UDP-GA) content and also by inhibiting the transferase activity [66].

The effects of piperine on UDP-glucose dehydrogenase (UDP-GDH) and glucuronidation potentials of rat and guinea pig liver and intestine were studied *in vitro*. Piperine caused a concentration-related strong inhibition of UDP-GDH (50% at 10 *μ*M) reversibly and equipotently, in both tissues. Partially purified rat liver UDP-GDH was used to obtain the kinetic values at pH optima of 9.4 and 8.6. At pH 9.4, *K*_*m*_ = 15 *μ*M, *V*_max⁡_ = 5.2 nmol, with NADH—*K*_*i*_ = 6 *μ*M, with NAD—*K*_*i*_ = 16 *μ*M were obtained. At pH 8.6, *K*_*m*_ = 35 *μ*M, *V*_max⁡_ = 7.5 nmol, and *K*_*i*_ = 15 *μ*M. In all of these cases, piperine caused noncompetitive inhibition. Data from structure activity comparisons of piperine analogs indicated that the presence of conjugated double bonds in the side chain of the molecule is a factor in piperine inhibition. However, the UDP-GA contents were decreased less effectively by piperine in isolated rat hepatocytes compared with enterocytes of guinea pig small intestine. Piperine at 50 *μ*M caused a marginal decrease of UDP-GA in hepatocytes when the rate of glucuronidation of 3-hydroxybenzo(a)pyrene (3-OH-BP) decreased by about 40%. The decrease obtained at 10 *μ*M piperine in intestinal cells was comparable to that obtained at 50–100 *μ*M in hepatocytes. UGT activities towards 3-OH-BP (UGT1A1) and 4-OH-biphenyl (UGT2B1) were also determined. Piperine did not affect the rate of glucuronidation of 4-OH-biphenyl in rat liver, whereas that of 3-OH-BP was impaired significantly. In guinea pig small intestine, both these activities were inhibited significantly requiring less than 25 *μ*M piperine to produce more than 50% inhibition of UGT(s). The results suggested that (i) piperine is a potent inhibitor of UDP-GDH, (ii) inhibition is offered exclusively by the conjugated double bonds of the molecule, and (iii) piperine exerts stronger effects on intestinal glucuronidation than in rat liver [16].

Piperine inhibits human P-glycoprotein and CYP3A4 (CYP: Cytochrome P450). Both the proteins are expressed in enterocytes and hepatocytes and contribute to a major extent to first-pass elimination of many drugs. This indicates that dietary piperine could affect plasma concentrations of P-glycoprotein and CYP3A4 substrates in humans, in particular if these drugs are administered orally. Some of the metabolizing enzymes inhibited or induced by piperine include CYP1A1, CYP1B1, CYP1B2, CYP2E1, and CYP3A4. Most of the drugs metabolized by these enzymes will therefore be influenced by bioenhancers [17].

#### 4.1.2. Drugs/Nutraceuticals Bioenhanced by Piperine

Piperine acts as an antimicrobial bioenhancer which enhances bioavailability and bioefficacy of drugs by acting on drug metabolism. It also acts as a nutritional bioenhancer which enhances bioavailability and absorption of nutrients by acting on gastrointestinal tract. Allameh et al. [68] reported that piperine enhances bioavailability of aflatoxin B_1_ in rat tissues. A 10 mg dose of piperine causes a marked increase in serum gonadotropins and a decrease in intratesticular testosterone concentration, despite normal serum testosterone titres in adult male albino rats [69]. However, some of the experimental findings also indicated the ability to decrease the bioavailability of drugs, like rifampicin [70, 71], isoniazid [72], and Diclofenac sodium [73].

Piperine has been shown to inhibit several cytochrome P450-mediated pathways and phase II reactions in animal models. Piperine, or mixtures containing piperine, has been shown to increase the bioavailability, blood levels, and efficacy of many of drugs (Table 3) and nutraceuticals (Table 4). Administration of piperinesignificantly increased plasma concentrations of rifampicin,phenytoin, spartein, sulfadiazine, tetracycline, propranolol, and theophylline in humans.

**Piperine and Phenytoin**

Effect of piperine on pharmacokinetics of phenytoin was studied in healthy volunteers. In a crossover study, five volunteers received either a single oral dose (300 mg) of phenytoin alone or in combination with multiple doses of piperine (20 mg × 7 days) followed by an oral dose of phenytoin. Blood samples were collected at 0.5, 1, 2, 3, 4, 8, 12, 24, and 48 h after drug administration and analyzed for phenytoin by the enzyme-multiplied immunoassay technique. The results showed that a single daily dose of piperine for 7 days decreased the *t*_1/2*α*_ (*P* < 0.05), prolonged the *t*_1/2_ (*P* < 0.01), and produced a higher AUC (*P* < 0.05) in comparison to phenytoin alone. It is therefore concluded that piperine on multiple-dose administration alters the pharmacokinetic parameters of the antiepileptic [76].

A preliminary pharmacokinetic study was carried out in mice by administering phenytoin (10 mg) orally, with or without piperine (0.6 mg). Subsequently, oral pharmacokinetics of phenytoin was carried out in six healthy volunteers in a crossover design. Phenytoin tablet (300 mg) was given 30 minutes after ingestion of a soup (*Melahu rasam*) with or without black pepper. A further study of intravenous pharmacokinetics of phenytoin (1 mg) in rats with or without oral pretreatment with piperine (10 mg) was also conducted. The phenytoin concentration in the serum was analyzed by HPLC. The study showed a significant increase in the kinetic estimates of *K*_*a*_, AUC_0−10_ and AUC_0−*∞*_ in the piperine fed mice. Similarly, in human volunteers piperine increased *K*_*a*_, AUC_0−48_, AUC_0−*∞*_ and delayed elimination of phenytoin. Intravenous phenytoin in the oral piperine-treated rat group showed a significant alteration in the elimination phase indicating its metabolic blockade [85].

The effect of piperine on oral bioavailability of phenytoin was studied in human volunteers. The objective of this study was to explore the effect of a single dose of piperine in patients with uncontrolled epilepsy on the steady-state pharmacokinetics of phenytoin. Two groups of 10 patients each receiving either a 150 mg or 200 mg twice daily dose of phenytoin were selected. 12 h after the night dose, venous blood samples were collected at 0, 0.5, 1, 2, 4, 6, 9, and 12 h after administration of phenytoin. On the next study day, piperine 20 mg was administered along with phenytoin and samples were collected similarly. There was a significant increase in AUC_0−12 h_ (*P* < 0.01), *C*_max⁡_ (*P* < 0.001), and *K*_*a*_ (*P* < 0.05), whereas the changes in *K*_el_ and *t*_max⁡_ were not significant. The results showed that piperine enhanced the bioavailability of phenytoin significantly, possibly by increasing the absorption [86].

**Piperine and Pentobarbitone**

Effect of piperine on pentobarbitone-induced hypnosis in rats was studied. Piperine treatment in rats, treated chronically with phenobarbitone, significantly potentiated pentobarbitone sleeping time, as compared to the controls. There was no alteration in barbital sodium sleeping time. It is possible that piperine inhibits liver microsomal enzyme system and thereby potentiates the pentobarbitone sleeping time [78].

**Piperine with Propranolol and Theophylline**

The effects of piperine on the bioavailability and pharmacokinetics of propranolol and theophylline were studied. Six subjects in each group received a single oral dose of propranolol (40 mg) or theophylline (150 mg) alone or in combination with piperine (20 mg) daily for 7 days. An earlier *t*_max⁡_ and a higher *C*_max⁡_ and AUC were observed in the subjects who received piperine and propranolol. It produced a higher *C*_max⁡_, longer *t*_1/2_, and a higher AUC with theophylline. In clinical practice, the enhanced systemic availability of oral propranolol and theophylline could be exploited to achieve better therapeutic control and improved patient compliance [77].

**Piperine and Nimesulide**

Influence of piperine on nimesulide-induced antinociception was studied in mice. The plasma concentration after oral administration of nimesulide (10 mg/kg) alone was 8.03 ± 0.99 *μ*g/mL. However, when it was administered with piperine (10 mg/kg), the plasma concentration of nimesulide increased to 11.9 ± 0.23 *μ*g/mL. This indicates that piperine inhibits the biotransformation and metabolism of nimesulide leading to significantly (*P* < 0.05) higher levels of drug in the systemic circulation. The findings of this study suggest that piperine could be used as a biological enhancer when coadministered with nimesulide [82].

**Piperine and Curcumin**

Influence of piperine on the pharmacokinetics of curcumin in animals and human volunteers was studied. The medicinal properties of curcumin obtained from *Curcuma longa* Linn. cannot be utilized because of poor bioavailability due to its rapid metabolism in the liver and intestinal wall. The effect of combining piperine, a known inhibitor of hepatic and intestinal glucuronidation, was evaluated on the bioavailability of curcumin in rats and healthy human volunteers. When curcumin was given alone, at dose 2 g/kg to rats, moderate serum concentrations were achieved over a period of 4 h. Concomitant administration of piperine 20 mg/kg increased the serum concentration of curcumin for a short period of 1-2 h after drug. *t*_max⁡_ was significantly increased (*P* < 0.02) while *t*_1/2_ and Cl_*B*_ significantly decreased (*P* < 0.02), and the bioavailability was increased by 154%. On the other hand, in humans after a dose of 2 g curcumin alone, serum levels were either undetectable or very low (Table 5). Concomitant administration of piperine 20 mg produced much higher concentrations from 0.25 to 1 h after drug (*P* < 0.01 at 0.25 and 0.5 h; *P* < 0.001 at 1 h); the increase in bioavailability was 200%. The study shows that in the dosages used, piperine enhances the serum concentration, extent of absorption, and bioavailability of curcumin in both rats and humans with no adverse effects [79].

**Piperine and *β*-Carotene**

The effectiveness of piperine was evaluated for its ability to improve serum response of *β*-carotene during oral supplementation using a double-blind, crossover study design. Subjects were randomly selected to ingest a daily *β*-carotene dose (15 mg) either with 5 mg of piperine or placebo during each of two 14-day supplementation periods. Intersubject variability in presupplementation serum *β*-carotene levels was minimized by limiting the selection of volunteers to healthy, adult males with fasting serum *β*-carotene values <20 pg/dL. The results indicate that significantly greater increases (*P* < 0.0001) in serum *β*-carotene occurred during supplementation with *β*-carotene plus piperine (49.8 ± 9.6 pg/dL versus 30.9 ± 5.4 pg/dL) compared to *β*-carotene plus placebo. Supplementation with betacarotene plus piperine for 14 days produced a 60% greater increase AUC than was observed during supplementation with *β*-carotene plus placebo (Table 6). Study suggests that the serum response during oral *β*-carotene supplementation is improved through the nonspecific, thermogenic property of piperine, described in this paper as thermonutrient in action [20].

**Piperine and Coenzyme Q_10_**

An extract from the fruits of black pepper consisting of a minimum of 98% pure piperine was evaluated in a clinical study using a double-blind design. The relative bioavailability of 90 mg and 120 mg of coenzyme *Q*_10_ administered in a single-dose experiment or in separate experiments for 14 and 21 days with placebo or with 5 mg of piperine was determined by comparing measured changes in plasma concentration. The intersubject variability was minimized by limiting the selection of individuals to healthy adult male volunteers with (presupplementation) fasting coenzyme *Q*_10_ values between 0.30 and 0.60 mg/L. The results of the single-dose study and the 14-day study indicate smaller, but not significant, increases in plasma concentrations of coenzyme *Q*_10_ in the control group compared with the group receiving coenzyme *Q*_10_ with a supplement of piperine. Supplementation of 120 mg coenzyme *Q*_10_ with piperine for 21 days produced approximately 30% greater AUC than was observed during supplementation with coenzyme *Q*_10_ plus placebo. It is postulated that the bioenhancing mechanism of piperine to increase plasma levels of supplemental coenzyme *Q*_10_ is nonspecific and possibly based on its description in the literature as a thermonutrient [106].

**Piperine and Rifampicin**

Piperine augments transcription inhibitory activity of rifampicin by several folds against *Mycobacterium smegmatis*. A 24 : 1 (w/w) mixture of antibiotic rifampicin and piperine shows remarkable growth inhibitory effect on *M. smegmatis*, and this inhibition is higher than that of rifampicin alone. Interestingly, piperine alone, even at higher concentration, does not inhibit the growth of *Mycobacterium*. As RNA polymerase is the site of action of rifampicin, the enzyme was purified from *M. smegmatis* and the mixture of rifampicin and piperine was found to abrogate nonspecific transcription catalyzed by *M. smegmatis *RNA polymerase. The effect is higher than rifampicin alone and piperine shows no effect independently. When RNA polymerase was purified from a rifampicin-resistant strain of *M. smegmatis*, the enzymatic activity, otherwise resistant to rifampicin, significantly decreases in the presence of piperine along with rifampicin. Piperine enhances the binding ability of rifampicin to RNA polymerase [88].

The amount of piperine used in the range of 0.4–0.9% by weight of the antituberculosis and antileprosy drugs. Antituberculosis composition contains rifampicin (100–300 mg), isoniazid (100–300 mg) and pyrazinamide (500–1000 mg) (combination of any two), and piperine (5–29 mg). Antileprosy composition contains rifampicin (100–300 mg), dapsone (50–100 mg), and piperine (5–20 mg). Effect of piperine on pharmacokinetic parameters of rifampicin, isoniazid, and pyrazinamide combination was studied in human volunteers. The results from Table 7 indicate that piperine significantly increases the bioavailability of antituberculosis drugs like rifampicin, isoniazid, and pyrazinamide [107, 108].

Risorine is a formulation developed by the Indian Institute of Integrative Medicine, Jammu, and marketed in India in November 2009 in public—private partnership with Cadila Pharmaceutical Ltd, Ahmedabad. Risorine has been approved for marketing by Drug Controller General of India, after successful completion of all the phased clinical trials. It contains rifampicin (200 mg), isoniazid (300 mg), and piperine (10 mg). It has been found to be bioequivalent with commercially available rifampicin preparations. This is due to enhanced uptake of the drug by body cells, and also because the drug remains available in blood for longer durations. Combining piperine with rifampicin decreases the dose of rifampicin from 450 to 200 mg [109].

**Piperine with Amoxicillin Trihydrate and Cefotaxime**

Effect of coadministration of piperine on pharmacokinetics of *β*-lactam antibiotics was studied in rats. Coadministration of piperine enhanced bioavailability of *β*-lactam antibiotics like amoxicillin trihydrate and cefotaxime sodium significantly in rats. The improved bioavailability is reflected in various pharmacokinetic parameters, namely *t*_max⁡_, *C*_max⁡_, *t*_1/2_, and AUC of these antibiotics. The increased bioavailability could be attributed to the effect of piperine on microsomal metabolizing enzymes or enzymes system [89].

**Piperine and Epigallocatechin Gallate (EGCG)**

Piperine enhanced the bioavailability of the tea polyphenol EGCG in Mice. EGCG, from green tea (*Camellia sinensis*), has demonstrated chemopreventive activity in animal models of carcinogenesis. Cotreatment with piperine enhanced the bioavailability of EGCG in mice. Intragastric coadministration of 163.8 *μ*mol/kg EGCG and 70.2 *μ*mol/kg piperine to male CF-1 mice increased the plasma *C*_max⁡_ and AUC by 1.3-fold compared to mice treated with EGCG only. Piperine appeared to increase EGCG bioavailability by inhibiting glucuronidation and gastrointestinal transit. Piperine (100 *μ*mol/L) inhibited EGCG glucuronidation in mouse small intestine (by 40%) but not in hepatic microsomes. Piperine (20 *μ*mol/L) also inhibited production of EGCG-3′′-glucuronide in human HT-29 colon adenocarcinoma cells. Small intestinal EGCG levels in CF-1 mice following treatment with EGCG alone had a *C*_max⁡_ = 37.50 ± 22.50 nmol/g at 60 min that then decreased to 5.14 ± 1.65 nmol/g at 90 min; however, cotreatment with piperine resulted in a *C*_max⁡_ = 31.60 ± 15.08 nmol/g at 90 min, and levels were maintained above 20 nmol/g until 180 min. This resulted in a significant increase in the small intestine EGCG AUC (4621.80 ± 1958.72 nmol/g/min versus 1686.50 ± 757.07 nmol/g/min). EGCG appearance in the colon and the feces of piperine-cotreated mice was slower than in mice treated with EGCG alone [90].

**Piperine and Oxytetracycline (OTC)**

The pharmacokinetic profile of orally administered oxytetracycline (10 mg/kg body weight) was studied 7 days after oral treatment of *P. longum* (15 mg equivalent/kg) in White Leghorn birds (WLH) (2.0–2.8 kg). On day 8, oxytetracycline (OTC) was administered orally and blood samples were collected from the wing vein in heparinized vials for plasma separation at 0 (before treatment), 15, 30, 60, 120, 240, 360, 480, and 600 min after OTC administration. Plasma OTC concentrations were determined by microbial assay technique using *Bacillus cereus* var. *mycoides* (ATCC 11778) as test organism. The plasma levels of OTC against time were adequately described by one compartment open model (Table 8). The pharmacokinetic data (Table 9) revealed that *P. longum*-treated animals had significantly higher AUC, AUMC, and MRT. Prior treatment of *P. longum* significantly reduced *β* and increased *t*_1/2_. The Cl_*B*_ reduced by 21%, whereas *t*_*d*_ increased by 29%. The treatment with *P. longum* reduced loading and maintenance dose by 33.3% and 39%, respectively [91].

**Piperine and Ciprofloxacin**

The influence of coadministration of piperine on pharmacokinetic profile of ciprofloxacin in rabbits was studied. The study reveals that piperine has affected statically to the pharmacokinetics of the ciprofloxacin which is significant with change in *C*_max⁡_, *t*_max⁡_, AUC, and *K*_el_. The results obtained are the combined effect of piperine on the absorption kinetics and the inhibition of the metabolism of the ciprofloxacin. This leads to increase bioavailability of ciprofloxacin when administered with piperine [92].

The potentiating effect of piperine with ciprofloxacin in *in vitro* combination against *Staphylococcus aureus *ATCC 29213 was studied and its suggestive role as an efflux pumps inhibitor. Piperine, in combination with ciprofloxacin, markedly reduced the MIC and mutation prevention concentration of ciprofloxacin for *S. aureus*, including methicillin resistant *S. aureus*. *In vitro* combination studies, the final concentrations ranged from 0.03 to 64 *μ*g/mL for ciprofloxacin and from 0.8 to 50 *μ*g/mL for piperine. The final bacterial inoculum in each well was 5 × 105 CFU/mL. The plates were incubated at 37°C for 24 h. Piperine did not show any antibacterial activity when tested up to 100 *μ*g/mL.  Table 10 is showing that there was a 2-fold reduction in the MIC of ciprofloxacin (from 0.25 to 0.12 *μ*g/mL) for *S*. *aureus* ATCC 29213 when tested in combination with piperine at 12.5 and 25 *μ*g/mL. The MIC was further reduced to 4-fold with 50 *μ*g/mL piperine [8]. Piperine inhibits the efflux of ethidium bromide and ciprofloxacin from bacterial cells (Table 11). It is a P-glycoprotein inhibitor that inhibits ciprofloxacin efflux [8].

**Piperine and Nevirapine**

Influence of piperine on the pharmacokinetics of nevirapine under fasting conditions was studied. This was a crossover, placebo-controlled pilot study conducted in a total of eight healthy adult males aged 20–40 years. Subjects were randomly assigned to receive piperine 20 mg or placebo each morning for 6 days, and on day 7, nevirapine (200 mg) plus piperine (20 mg) or nevirapine plus placebo in a crossover fashion. Blood samples were collected from 1–144 h after dose for pharmacokinetic analysis. Mean *C*_max⁡_, AUC_*t*_, AUC_0−*∞*_, and *C*_last_ values of nevirapine were increased by approximately 120%, 167%, 170%, and 146%, respectively, when coadministered with piperine. This pilot study provided evidence for enhanced bioavailability of nevirapine when administered with piperine [93].

**Piperine with Diclofenac Sodium and Pentazocine**

The analgesic activity of *P. nigrum* extract *per se* and its interaction with diclofenac sodium and pentazocine in albino mice was studied. Healthy albino mice of either sex weighing 25–30 grams were taken and divided into 4 groups of 8 animals each. Peripheral analgesic activity was evaluated by acetic acid-induced writhing test, using diclofenac sodium (5 mg/kg), *P. nigrum* extract (10 mg/kg), and their combination (5 + 10 mg/kg) orally. Similarly central analgesic activity was studied using tail flick method and pentazocine (5 mg/kg) orally was used instead of diclofenac sodium. *P. nigrum* extract alone did not show any significant analgesic activity in tail flick and writhing methods. However, results of acetic acid-induced writhing model showed that diclofenac sodium reduces writhing 54.90% with respect to control when administered alone, but showed significant decrease in writhes 78.43% with respect to control when *P. nigrum* extract was coadministered with diclofenac sodium. When *P. nigrum* extract combined with pentazocine showed significant increase in tail flick latency in comparison with pentazocine alone and control group (*P* < 0.05), the results suggest that the *P. nigrum* extract significantly increased the analgesic activity of diclofenac sodium and pentazocine [47].

**Piperine and Pefloxacin**

The pharmacokinetics of orally administered pefloxacin were studied to evaluate the bioenhancing effect of the herbal bioenhancer, trikatu, in mountain Gaddi goats (*n* = 6). The findings of the study revealed a decreased plasma concentration (*P* > 0.05) of pefloxacin following trikatu administration during the absorption phase (10, 15, and 20 min after pefloxacin administration). In contrast, the plasma concentrations of pefloxacin were significantly higher at 4, 6, 8, and 12 h (during the elimination phase) of the pefloxacin administration. The findings of the investigation revealed higher values for AUC, AUMC, MRT, *t*_*d*_, and bioavailability. Trikatu treatment, however, significantly reduced *t*_1/2_ and zero time intercept of the elimination phase. The *V*_*d*(area)_ and *V*_*d*(*B*)_ were significantly higher in trikatu-treated animals indicating a better penetration of the drug. Based on the MIC of 0.8 *μ*g/mL of pefloxacin; a priming dose of 6.0 mg/kg and a maintenance dose of 2.21 mg/kg are required to be administered at 8 h intervals. For practical purposes in goats this would mean a priming dose of 6 mg/kg and a maintenance dose of 2 mg/kg given by the oral route to be repeated at 8 h intervals [94].

**Piperine with Ampicillin and Norfloxacin**

Effect of piperine on oral bioavailability of ampicillin and norfloxacin was studied in rabbits. Coadministration of piperine (20 mg/kg), an alkaloid from *P. nigrum*, enhanced oral bioavailability of ampicillin and norfloxacin in animal model. This is reflected in various pharmacokinetic measurements like *C*_max⁡_, *t*_max⁡_, AUC, and *t*_1/2_ of the above antibiotics in animal model shown in Tables 12, 13, and 14 [95].

**Piperine and Carbamazepine**

The effect of piperine on the steady-state pharmacokinetics of a single dose of carbamazepine in poorly controlled epilepsy patients on carbamazepine monotherapy was studied. Patients (*n* = 10 each) receiving either 300 mg or 500 mg dose of carbamazepine twice a day were selected. After administration of carbamazepine, venous blood samples were collected at 0, 0.5, 1, 2, 4, 6, 9, and 12 h. Subsequently, piperine (20 mg *p.o.*) was administered along with carbamazepine and samples were collected similarly. Piperine significantly increased the mean plasma concentrations of carbamazepine at most of the time points in both dose groups. There was a significant increase in AUC_0−12 h_ (*P* < 0.001), average C_ss_ (*P* < 0.001), *t*_1/2_ (*P* < 0.05), and a decrease in *K*_el_ (*P* < 0.05), in both the dose groups, whereas changes in *K*_*a*_ and *t*_1/2*α*_ were not significant. *C*_max⁡_ (*P* < 0.01) and *t*_max⁡_ (*P* < 0.01) were increased significantly following piperine administration in the 500 mg dose group; however, these parameters were not significant in the lower dose group. Piperine could significantly enhance the oral bioavailability of carbamazepine, possibly by decreasing the elimination or by increasing its absorption [96].

**Piperine and Fexofenadine**

Effect of piperine, a major component of black pepper, on the oral exposure of fexofenadine in rats was studied. Pharmacokinetic parameters of fexofenadine were determined in rats following an oral (10 mg/kg) administration of fexofenadine in the presence and absence of piperine (10 or 20 mg/kg). Compared to the control group given fexofenadine alone, the combined use of piperine increased the AUC of fexofenadine by 180% to 190% while there was no significant change in *C*_max⁡_ and *t*_1/2_ of fexofenadine in rats. The bioavailability of fexofenadine was increased by approximately 2-fold via the concomitant use of piperine. Furthermore, *t*_max⁡_ tends to be increased which might be attributed to the delayed gastric emptying in the presence of piperine. In conclusion, piperine significantly enhanced the oral exposure of fexofenadine in rats likely by the inhibition of P-glycoprotein-mediated cellular efflux during the intestinal absorption, suggesting that the combined use of piperine or piperine-containing diet with fexofenadine may require close monitoring for potential drug-diet interactions [97].

**Piperine and Metronidazole**

Effect of piperine on bioavailability of metronidazole was studied in rabbits. Male New Zealand white rabbits (2.0–2.5 kg body weight) used for pharmacokinetic and bioavailability study. Three groups of rabbits were formed from which one group was considered as control and received only vehicle (distilled water) orally. The remaining two groups were treated with metronidazole and combination of metronidazole and piperine, respectively. About 1 mL of blood sample was collected at the different time intervals and analyzed spectrophotometrically by HPLC. Different pharmacokinetic parameters have shown to enhance bioavailability of metronidazole in combination with piperine than metronidazole alone (Table 15). So, it is concluded that bioavailability of metronidazole was significantly enhanced in the presence of piperine [98].

**Piperine and Resveratrol**

Effect of piperine on oral bioavailability of resveratrol was studied in mice. Employing a standardized LC-MS assay, the effect of piperine coadministration with resveratrol on serum levels resveratrol and resveratrol-3-*O*-*β*-D-glucuronide in C57BL mice was determined. Mice were administered resveratrol (100 mg/kg) or resveratrol (100 mg/kg) + piperine (10 mg/kg), and the serum levels of resveratrol and resveratrol-3-*O*-*β*-D-glucuronide were analyzed at different times. The AUC to resveratrol was enhanced to 229% and the *C*_max⁡_ was increased to 1544% with the addition of piperine. Study demonstrated that piperine significantly improves the *in vivo* bioavailability of resveratrol [100].

**Piperine and Gatifloxacin**

Influence of coadministration of piperine on pharmacokinetic profile of gatifloxacin was studied in layer birds. The pharmacokinetic profile (Tables 16 and 17) of gatifloxacin (10 mg/kg body weight) along with piperine coadministration (15 mg/kg) via single oral dose in layer birds showed *t*_1/2_, *C*_max⁡_, and AUC of 4.03 ± 0.097 h, 2.14 ± 0.019 *μ*g/mL, and 17.54 ± 0.204 *μ*g·h/mL, respectively, which is found significantly higher than gatifloxacin alone (3.74 ± 0.073 h, 1.74 ± 0.023 *μ*g/mL, and 15.25 ± 0.219 *μ*g·h/mL, resp.). This study reveals that piperine has significant effect on the pharmacokinetics of the gatifloxacin. There is enhancement in bioavailability (*F*) from 74.52 ± 1.021% to 85.74 ± 0.956% in piperine + gatifloxacin-treated group. The results obtained are the combined effect of piperine on the absorption kinetics and the inhibition of the metabolism of gatifloxacin [101].

The influence of coadministration of piperine on pharmacokinetic profile of gatifloxacin was studied in broiler birds. The pharmacokinetic profile of gatifloxacin (10 mg/kg body weight) along with piperine coadministration (15 mg/kg) via single dose oral administration in broiler birds showed *t*_1/2_, *C*_max⁡_, and AUC of 4.1 ± 0.092 h, 2.14 ± 0.02 *μ*g/mL, and 17.58 ± 0.17 *μ*g·h/mL, respectively, which is found significantly higher than *t*_1/2_, *C*_max⁡_ and AUC of 3.81 ± 0.07 h, 1.74 ± 0.024 *μ*g/mL, and 15.24 ± 0.23 *μ*g·h/mL, respectively, obtained after single oral administration of gatifloxacin (10 mg/kg body weight) alone. This study reveals that piperine has significant effect on the pharmacokinetics of the gatifloxacin. There is significant enhancement of bioavailability (*F*) in piperine-treated group (81.95 ± 1.56%) compared to gatifloxacin alone treated group (72.96 ± 1.10%). The results obtained are the combined effect of piperine on the absorption kinetics and the inhibition of the metabolism of gatifloxacin [102].

**Piperine and Atenolol**

A bioavailability of atenolol in presence of piperine was studied in male New Zealand white rats. All rats were divided in three groups. First group was considered as control and received only vehicle (distilled water) orally. The remaining two groups were treated with atenolol and combination of atenolol and piperine, respectively. About 2 mL of blood sample was collected at the different time intervals and analyzed spectrophotometrically by HPLC. The pharmacokinetic parameters obtained after administration of atenolol (100 mg/kg) and combination of atenolol (100 mg/kg) and piperine (10 mg/kg). So, from Table 18 it can be concluded that bioavailability of atenolol was significantly enhanced in presence of piperine [103].

**Piperine and Ibuprofen**

Influence of piperine on ibuprofen-induced antinociception and its pharmacokinetics was studied. Piperine (10 mg/kg) significantly increased the dose-dependent antinociceptive activity of ibuprofen evaluated by both acetic acid writhing and formalin test, when it was administered with ibuprofen. Ibuprofen plasma concentration was also increased when it was administered with piperine. The synergistic antinociception activity of ibuprofen when administered with piperine can be attributed to increased plasma concentration of ibuprofen. From this study it can be concluded that piperine can be used as a bioenhancer along with ibuprofen [104].

**Piperine and Losartan Potassium**

Effect of piperine on bioavailability of losartan potassium was studied in male New Zealand white rats. All rats were divided in three groups. First group received only mineral water orally. Second group received losartan potassium (100 mg/kg). Third group received losartan potassium (100 mg/kg) and piperine (10 mg/kg). About 2 mL of blood sample was collected at the different time intervals and analyzed by HPLC. The pharmacokinetic parameters obtained after administration of losartan potassium (100 mg/kg) and combination of losartan potassium (100 mg/kg) and piperine (10 mg/kg). So, from Table 19 it can be concluded that bioavailability of losartan potassium was significantly enhanced in presence of piperine [105].

### 4.2. *Zingiber officinale*

The major pungent compounds in *Zingiber officinale* (Ginger) of rhizome extract contain potentially active gingerols, which can be converted to shogaols, zingerone, and paradol [110, 111]. The odor of ginger depends mainly on its volatile oil, the yield of which varies from 1 to 3% [112]. In laboratory animals, the gingerols increase the motility of the gastrointestinal tract and have analgesic, sedative, antipyretic, and antibacterial properties [113]. [6]-gingerol is the major pungent principle of ginger. The chemopreventive potentials of [6]-gingerol present a promising future alternative to expensive and toxic therapeutic agents [114]. Ginger exhibits activities like antiulcer activity [115, 116], antithrombotic activity [117], antimicrobial activity [118], antifungal activity [119], anti-inflammatory activity [117, 120–122], antidiabetic activity [123], antiemetic activity [124, 125], anthelmintic activity [126], analgesic and antipyretic activity [121], antioxidant and antiapoptotic activity [122], and anticancer activity [127].

Ginger acts powerfully on GIT mucous membrane. The role of ginger is to regulate intestinal function to facilitate absorption. The composition containing *Z. officinale* alone provides bioavailability/bioenhancing activity in the range of 30–75%, and piperine and *Z. officinale*, and provides the bioavailability of drugs in the range of 10–85%. The dosage of bioenhancer from *Z. officinale* as extract is in the range of 10–30 mg/kg body weight and piperine is in the range of 4–12 mg/kg body weight. The dosage of bioenhancer from *Z. officinale* as bioactive fraction is in the range of 5–15 mg/kg body weight, preferably 30 mg/kg body weight and piperine is in the range of 6–10 mg/kg body weight, preferably 8 mg/kg body weight. The extracts or its fractions either in presence or absence of piperine have been found to be highly selective in their bioavailability enhancing activity (Table 20). It varies from almost nearly significant (20%) to highly significant (200%) [128, 129].

### 4.3. Niaziridin

Niaziridin is a nitrile glycoside that has been isolated from the leaves, pods, and bark of Drumstick (*Moringa oleifera*) [130]. *M. oleifera* has shown to exhibit activities like antifertility effect [131], antimicrobial activity [132], diuretic activity [133], anticancer activity [134, 135], anti-inflammatory activity [133, 135], hypotensive and spasmolytic activity [136], antifungal activity [137], antiulcer activity [138], antioxidant activity [139, 140], hepatoprotective activity [141], hypolipidaemic activity [142], antiteratogenic activity [143], and antiarthritic activity [144].

It enhances bioactivity of commonly used antibiotics such as rifampicin, ampicillin, tetracycline, and nalidixic acid against Gram-positive bacteria like *M. smegmatis *and *Bacillus subtilis *and Gram-negative bacteria like *E. coli* (Table 21). It enhances activity of commonly used antibiotics such as rifampicin, ampicillin, tetracycline, and nalidixic acids against *E. coli* Gram-negative bacteria. It enhances activity of rifampicin, ampicillin, tetracycline, and nalidixic acids by 1.2–19-fold against the Gram-positive strains. It enhances the activity of azole antifungal drugs such as clotrimazole against *Candida albicans* by 5-6-fold. However, the antifungal activity enhancement was observed only at a relatively higher concentration (10 *μ*g/mL) of the compound. It also facilitates the uptake of nutrients like Vitamin B_12_ through the intestinal gut membrane in combination, thus also functioning as bioavailability enhancer [145, 146].

### 4.4. Glycyrrhizin

Glycyrrhizin is a glycoside obtained from roots and stolon of Liquorice (*Glycyrrhiza glabra*). It has expectorant action to treat bronchitis and can also reduce inflammation, allergies, asthma, gastritis, peptic ulcers, rheumatism, and sore throat. It helps the liver to detoxify drugs and is used for treatment of liver disease. It strengthens the immune system, stimulates the adrenal gland, and is diuretic and laxative. Glycyrrhizin is 50 times sweeter than sugar. Primary uses include treatment for peptic ulcers and stomach ailments, respiratory, and intestinal passages. Glycyrrhizin exhibits activities like antihepatotoxic activity [147, 148], anti-inflammatory activity [149, 150], anticancer activity [151], and antiviral activity [152, 153].

The concentration of glycyrrhizin ranges from 0.05 to 50% of the weight of the antibacterial compounds. The concentration of glycyrrhizin ranges from 0.10 to 10% of the weight of the nutraceutical compounds. The concentration of glycyrrhizin ranges from 0.25 to 20% of the weight of the antifungal agents. The level of glycyrrhizin ranges from 10 to 10,000-fold of the weight of the anticancer compound used [154, 155].

Glycyrrhizin-mediated enhancement in the cell division inhibitory action of anticancer agent “Taxol” (paclitaxel) in the animal cell culture experiments using cancerous cell line MCF-7. The anticancerous activity of Taxol in terms of inhibiting the growth and multiplication of MCF-7 cancer cells was markedly enhanced by 5-fold. The cancerous cells growth inhibition by Taxol (0.01 *μ*g/mL) in presence of glycyrrhizin (1 *μ*g/mL) was higher than even the treatment with Taxol (0.05 *μ*g/mL) alone [154, 155]. 

It enhances bioactivity of commonly used antibiotics such as rifampicin, ampicillin, tetracycline, and nalidixic acids against Gram-positive bacteria like *M. smegmatis *and *Bacillus subtilis *and Gram-negative bacteria like *E. coli *(Table 22). It enhances activity of commonly used antibiotics such as rifampicin, ampicillin, tetracycline, and nalidixic acids against *E. coli* Gram-negative bacteria. It enhances activity of rifampicin, tetracycline, and nalidixic acids against the Gram-positive strains. It enhances the activity of azole antifungal drugs such as clotrimazole against *Candida albicans *[154, 155].

### 4.5. *Cuminum cyminum*

The main components of *C. cyminum* oil are *p*-mentha-1,4-dien-7-al, cumin aldehyde, *γ*-terpinene, and *β*-pinene [156]. *C. cyminum *exhibits activities like estrogenic activity [157], hypolipidaemic activity [158], antinociceptive and anti-inflammatory activity [159], anticonvulsant effect [160], anticancer activity [161], antimicrobial activity [156, 162], antitussive effect [163], antioxidant activity [164], and antifungal activity [165]. The doses of its fractions responsible for the bioavailability enhancement activity ranged from 0.5 to 25 mg/kg body weight. The dosage level of the composition comprising *C. cyminum *extract is in the range of 10–30 mg/kg body weight and composition comprising bioactive fraction is in the range of 2–20 mg/kg body weight. The composition contain *C. cyminum *extract or the fractions there of which provides bioavailability/bioenhancing activity in the range of 25–335% (Table 23). Polar and nonpolar extract of parts of *C. cyminum *and piperine increased bioavailability in the range of 25–435% [166, 167].

### 4.6. *Carum carvi*

*Carum carvi* (Caraway) contains caraway oil obtained from dried and crushed seeds. Carvone and limonene are the chief constituents of the oil and its odour and flavour are mainly attributed to them [168]. *C. carvi* exhibits activities like antiulcer effects [169], hypoglycemic effect [170], antimicrobial activity [156, 171], diuretic activity [172], antioxidant activity [171, 173], antiaflatoxigenic activity [174], and antifungal activity [171]. The effective dose of the bioenhancer extract is in the range of 5–100 mg/kg body weight and the dose bioactive fraction of bioenhancer is in the range of 1–55 mg/kg body weight. It has been reported to enhance bioavailability of antibiotics, antifungal, antiviral, and anticancerous drug. The extract or its fractions were found to be 20–110% more active and when used in combination with *Z. officinale,* it was found to be more effective, in the range of 10–150 mg/kg body weight. *C. carvi *in different combinations showed pronounced activity ranging from 25 to 95% in presence of piperine and amount of piperine in formulations ranged from 3 to 15 mg/kg body weight [5, 175]. The percent enhancement of bioavailability of different class of drugs by fractions of *C. carvi* and in combination with piperine and *Z. officinale *is shown in Table 24.

### 4.7. Allicin

Allicin is an allyl sulfur containing compound obtained from Garlic (*Allium sativum*). Allicin exhibits activities like antiplatelet activity [176], antioxidant activity [177, 178], antibacterial activity [179, 180], anticancer activity [181], Immunomodulating effect [182], antidiabetic activity [183, 184], antiparasitic activity [185], antifungal activity [186–188], antioxidant and anti-inflammatory activity [189, 190], and antiviral activity [191].

#### 4.7.1. Allicin and Cu^2+^

Cu^2+^ showed a dose-dependent fungicidal activity against *Saccharomyces cerevisiae* cells, and its lethal effect was extremely enhanced in the presence of allicin, an allyl sulfur compound from garlic. The fungicidal activity of Cu^2+^ was unaffected or rather attenuated by other sulfur-containing compounds such as *N*-acetyl-cysteine, l-cysteine, or dithiothreitol. Ca^2+^ could absolutely protect against the lethal effect of Cu^2+^ itself, but showed no protection against the fungicidal activity of Cu^2+^ newly generated in combination with allicin. Cu^2+^ accelerated an endogenous generation of reactive oxygen species (ROS) in *S. cerevisiae* cells at a lethal concentration, but such intracellular oxidative stress induction was not observed during cell death progression upon treatment with Cu^2+^ and allicin. A surfactant, sodium *N*-lauroyl sarcosinate (SLS), enhanced the solubilization of a few proteins including alkyl hydroperoxide reductase 1 (AHP1) in intact cells, accounting for the absence of this protein in the extract from allicin-treated cells. Allicin-treated cells were rendered extremely sensitive to the subsequent Cu^2+^ treatment as in the case of SLS-treated cells. Allicin-treated cells and SLS-treated cells similarly showed an increased sensitivity to exogenously added *tert*-butyl hydroperoxide (*t*-BOOH), organic peroxide that is detoxified by the action of AHP1. Our study suggests that allicin influences the mode of cell surface localization or the related function of AHP1 as a defense against phospholipid peroxidation by the external action of Cu^2+^ [192].

#### 4.7.2. Allicin and Amphotericin B

Enhancement of the fungicidal activity of amphotericin B (AmB) by allicin, an allyl-sulfur compound from garlic, against the yeast *Saccharomyces cerevisiae* as a model system was studied. AmB is a representative antibiotic for the control of serious fungal infections, and its fungicidal activity was greatly enhanced by allicin (Table 25). In addition to the plasma membrane permeability change, AmB induced vacuole membrane damage so that the organelles were visible as small discrete particles. Although allicin was ineffective in promoting AmB-induced plasma membrane disability, this compound enhanced AmB-induced structural damage to the vacuolar membrane even at a nonlethal dose of the antibiotic. Allicin could also enhance the antifungal activity of AmB against the pathogenic fungus *C. albicans *and against* Aspergillus fumigatus*. In contrast, allicin did not enhance the cytotoxic activity of AmB against cells of human promyelocytic leukemia (HL-60), a vacuole-less organism [187].

Allicin was found to inhibit ergosterol transport from the plasma membrane to the cytoplasm, which is considered to be a cellular protective response to AmB-induced vacuole disruption in *S. cerevisiae*. The study suggests that AmB lethality against *C. albicans* depends at least in part on its vacuole disruptive activity under the physiological condition permissive for invasive growth of the fungus [188].

Ogita et al. [193] reported that allicin enhances AmB-induced vacuole membrane damage by inhibiting ergosterol trafficking from the plasma membrane to the vacuole membrane. Ogita et al. [194] suggested that the fungicidal activity of AmB combined with allicin is involved in vacuole disruption but not in potassium ion efflux, and that the expression of allicin-mediated activity of AmB requires the presence of ergosterol in the plasma membrane.

### 4.8. Lysergol

Lysergol, a phytomolecule, is obtained from Morning Glory Plant (*Ipomoea spp.*) which enhances the killing activities of different antibiotics on bacteria and is a promising herbal bioenhancer. It has been isolated from higher plants like *Rivea corymbosa, Ipomoea violacea, *and* Ipomoea muricata*. Bioenhancing activities of lysergol are under investigation. The effective amount of lysergol as a bioenhancer and bioavailability enhancer is in the range of 1–10 *μ*g/mL but preferable dosage level is 10 *μ*g/mL. It enhances bioavailability of different drugs like rifampicin, tetracycline, and ampicillin. Lysergol enhances the antimicrobial effect of the antibiotic compound in the range of 2–12-fold (Table 26). It is effective against broad spectrum microbes, Gram-positive and Gram-negative, consisting *E. coli *(ATCC 10536), *B. subtilis *(ATCC 6051),* M. smegmatis *(ATCC 14468), and other similar microbes [2]. Patil et al. [195] reported that lysergol improved the bioavailability of berberine after oral administration in Sprague-Dawley rats.

### 4.9. *Aloe Vera* Gel and Whole Leaf Extract

*Aloe vera* had a salutary effect on both vitamin C and vitamin E. *Aloe vera* gel and whole leaf extracts shown increased plasma concentration and improved absorption of both, vitamin C and vitamin E. The *Aloe vera* gel extract was especially effective in slowing down and increasing the absorption of ascorbate. It prolonged its plasma concentration significantly, even for 24 h, and following an overnight fast. Both the gel and whole leaf extracts improved the absorption of vitamin E and prolonged its plasma concentration, especially after 8 h. AUC for vitamin C and vitamin E with Aloe gel and whole leaf extract is shown in Table 27. *Aloe vera* is unique in its ability to increase bioavailability of both of these vitamins and should be considered as a future nutritional herbal bioenhancer [196].

### 4.10. *Stevia rebaudiana*

*Stevia rebaudiana *(Stevia) is known as honey leaf which has been used as sweetener in South America. The chief constituent of stevia is stevioside, the glycoside which is 200 times sweeter than sucrose. Other constituents include steviol, austroinulin, rebaudioside, and dulcoside A. Extracts/fractions/pure isolates of stevia either alone or in combination with piperine are selective in enhancing the bioavailability/bioefficacy of drugs, nutraceuticals, and herbal drugs/formulations. The percentage of stevia in the bioenhancing composition varies from 0.01 to 80%. The dosage of bioenhancer derived from stevia extract is in the range of 0.01–50 mg/kg body weight and piperine is in the range of 0.01–12 mg/kg body weight. The dosage of bioenhancer derived from stevia bioactive fraction or pure compound is in the range of 0.01–40 mg/kg body weight, preferably 30 mg/kg body weight and piperine is in the range of 0.01–10 mg/kg body weight, preferably 8 mg/kg body weight. The dosage of bioenhancer derived from stevia leaf is in the range of 0.01–250 mg/dose of the drug, nutraceutical, or herbal extract. The dosage of bioenhancer derived from stevia fraction or pure compound is in the range of 0.01–75 mg irrespective of the amount of drug in the composition, preferably 1–30 mg/dose of the drug, nutraceutical, or herbal extract. Stevia enhanced bioavailability of different groups like antibiotics, antiobese drugs, antidiabetic drugs, antifungal drugs, antiviral drugs, anticancer drugs, cardiovascular drugs, anti-inflammatory, antiarthritic agents, antituberculosis/antileprosy drugs, anthelmintic/respiratory drugs, immune-modulators, antiulcer drugs, and herbal products or drugs [197].

### 4.11. Curcumin

Curcumin is the principal curcuminoid of the popular Indian spice turmeric (*Curcuma longa*). Curcumin suppresses drug metabolizing enzymes (CYP3A4) in the liver [198] as well as inducing changes in the drug transporter P-glycoprotein, hence increasing the *C*_max⁡_ and AUC of celiprolol and midazolam in rats [199]. The influence of curcumin before treatment on pharmacokinetic disposition of norfloxacin was studied in rabbits after single oral administration. The pharmacokinetic data revealed that curcumin-treated animals had significantly (*P* ≤ 0.05) higher AUC and AUMC. Prior treatment of curcumin significantly (*P* ≤ 0.05) increased *t*_1/2*β*_ and *V*_*d*_ of norfloxacin. Further treatment with curcumin reduced loading and maintenance doses by 26% and 24%, respectively [200].

### 4.12. Sinomenine

Sinomenine is an alkaloid obtained from *Sinomenium acutum, *widely used for treatment of rheumatic and arthritic diseases in China and Japan.

A single dose of paeoniflorin (150 mg/kg) alone and with sinomenine hydrochloride (90 mg/kg) was administered by gastric gavage to unrestrained conscious male Sprague-Dawley rats (250–300 g). A Blood sample was collected periodically via a jugular vein before and after dosing from 10 min to 12 h. HPLC assay was developed to determine the plasma concentrations of paeoniflorin. After coadministration of sinomenine, the *C*_max⁡_ of paeoniflorin was elevated, *t*_max⁡_ was delayed, AUC_0−*∞*_was increased, MRT was prolonged, CL_*B*_ was decreased, and *V*_*d*_ was reduced. These results from Table 28 indicate that sinomenine hydrochloride significantly improved the bioavailability of paeoniflorin in rats [201].

Sinomenine at 16 and 136 *μ*M concentrations could significantly enhance the absorption of paeoniflorin (20 *μ*M) by 1.5- and 2.5-fold, respectively, and subsequently improve the bioavailability of paeoniflorin in rats. The mechanism underlying the improvement of paeoniflorin's bioavailability was proposed that sinomenine could decrease the efflux transport of paeoniflorin by P-glycoprotein [202].

### 4.13. Genistein

Genistein is an isoflavone found in a number of dietary plants like soybean (*Glycine max*) and kudzu (*Pueraria lobata*) and has been studied for a number of potential health effects including anticancer and anti-inflammatory activity [203]. It is a well-known phytoestrogen [204].

#### 4.13.1. Genistein and Paclitaxel

The effect of orally administered genistein on the pharmacokinetics of paclitaxel administered through oral and intravenous route in rats was studied. A single dose of paclitaxel was administered orally (30 mg/kg) or intravenous route (3 mg/kg) alone or 30 min after oral administration of genistein (3.3 mg/kg or 10 mg/kg). The presence of 10 mg/kg genistein significantly (*P* < 0.05) increased the AUC (54.7%) of orally administered paclitaxel, which was due to the significantly (*P* < 0.05) decreased CL_F_ of paclitaxel (35.2%). Genistein also increased *C*_max⁡_ of paclitaxel significantly (*P* < 0.05 by 3.3 mg/kg, 66.8%; *P* < 0.01 by 10 mg/kg, 91.8%). Consequently, the *AB* of paclitaxel in the presence of genistein was 0.020–0.025, which was elevated more than the control group (0.016); the *RB* of orally administered paclitaxel was increased from 1.26 to 1.55-fold. 10 mg/kg genistein also significantly (*P* < 0.05) increased the AUC (40.5%) and reduced the CL_*B*_ (30%) of intravenously administered paclitaxel. The presence of genistein improved the systemic exposure of paclitaxel in this study [205].

#### 4.13.2. Genistein and Epigallocatechin Gallate

Cotreatment of HT-29 human colon cancer cells with genistein increased cytosolic EGCG by 2-5-fold compared with treatment with EGCG only. Intragastric coadministration of EGCG (75 mg/kg) and genistein (200 mg/kg) to CF-1 mice resulted in an increase in *t*_1/2_ (148.7 ± 16.4 versus 111.5 ± 23.4 min) and AUC (183.9 ± 20.2 versus 125.8 ± 26.4 mg/mL at 3 min) of EGCG. Cotreatment with genistein also increased the *C*_max⁡_, 6 h exposure AUC, and *t*_1/2_ of EGCG in the small intestine by 2.0-, 4.7-, and 1.4-fold, respectively, compared with mice treated with EGCG only. This study demonstrates that genistein can enhance EGCG bioavailability [206].

### 4.14. *Ammannia multiflora*

The methanolic extract of *Ammannia multiflora* (Lythraceae) showed significant bioenhancing activity with the antibiotic nalidixic acid. *A. multiflora* contains a novel compound ammaniol along with other compounds. The methanolic extract of *Ammannia multiflora* bioenhancing activity in combination with nalidixic acid against the two strains, CA8000 and DH5*α* of *Escherichia coli*. The results showed that the methanolic extract of *A*.*multiflora* possessed significant bioenhancing activity and reduced the dose of nalidixic acid fourfold. The methanolic extract of *Ammannia multiflora* was also tested for antimycobacterial activity against *Mycobacterium* H37Rv and was found to show moderate activity (MIC 25 *μ*g/mL) against this pathogen [207].

### 4.15. Capsaicin

Capsaicin is the active component of chili peppers (*Capsicum annum*). It is an irritant for mammals, including humans, and produces a sensation of burning in any tissue with which it comes into contact. Cruz et al. [208] reported that *Capsicum annum* reduces bioavailability of aspirin after oral administration in rats. Lopez et al. [209] have shown that capsaicin has little or no impact on bioavailability of ciprofloxacin.

Absorption and bioavailability of theophylline from a sustained release gelatin capsule were investigated in 10 male rabbits after oral administration (20 mg/kg), with and without a ground capsicum fruit suspension. Comparison of pharmacokinetic parameters showed that the concomitant absorption of capsicum increases AUC from 86.06 ± 9.78 mg·h/L to 138.32 ± 17.27 mg·h/L, *P* < 0.001, *C*_max⁡_ from 6.65 ± 0.76 to 8.78 ± 0.98 mg/L, *P* < 0.01, and MRT from 14.94 ± 2.97 h to 20.98 ± 5.75 h, *P* < 0.001. A second administration of the capsicum suspension, 11 hours after dosing, produced a new rise of theophylline plasma levels in every rabbit. This indicated that due to action of capsaicin bioavailability of theophylline was enhanced [210].

## 5. Other Bioenhancers

### 5.1. Quercetin

Quercetin is a plant-derived flavonoid found in fruits, vegetables, leaves, and grains. Quercetin has exhibited activities including antioxidant, radical scavenging, anti-inflammatory, antiatherosclerotic, anticancer, and antiviral effects [211]. Quercetin is an inhibitor of CYP3A4 and a modulator of P-glycoprotein [212–215]. It has been shown to decrease bioavailability of cyclosporin in pigs and rats [212]. Wang et al. [216] reported that coadministration of quercetin with digoxin leads to lethal effects in pigs.

#### 5.1.1. Quercetin and Paclitaxel

The effect of quercetin on the bioavailability of paclitaxel was studied after oral administration in rats. Paclitaxel (40 mg/kg) and prodrug (280 mg/kg, 40 mg/kg as the paclitaxel) were administered orally to rats pretreated with quercetin (2, 10, 20 mg/kg). The pharmacokinetic parameters shown in Table 29 indicated that quercetin increases bioavailability of paclitaxel in rats [217].

#### 5.1.2. Quercetin and Verapamil

Pharmacokinetic parameters of verapamil and norverapamil were determined after the oral administration of verapamil (10 mg/kg) to rabbits in the presence and absence of quercetin (5.0 and 15 mg/kg). While coadministration of quercetin concurrently was not effective to enhance the oral exposure of verapamil, pretreatment of quercetin 30 min before verapamil administration significantly altered the pharmacokinetics of verapamil. Compared with the control group, the *C*_max⁡_ and AUC of verapamil increased approximately 2-fold in the rabbits pretreated with 15 mg/kg quercetin. There was no significant change in *t*_max⁡_ and *t*_1/2_ of verapamil in the presence of quercetin. Consequently, absolute and relative bioavailability values of verapamil in the rabbits pretreated with quercetin were significantly higher (*P* < 0.05) than those from the control group. In conclusion, pretreatment of quercetin significantly enhanced the oral exposure of verapamil [218].

#### 5.1.3. Quercetin and Diltiazem

The effect of quercetin (2, 10, 20 mg/kg) pretreatment on the bioavailability of diltiazem (15 mg/kg) was studied in rabbits after oral administration. The plasma concentrations of diltiazem in the rabbits pretreated with quercetin were increased significantly (*P* < 0.05, at 2 mg/kg; *P* < 0.01, at 10 and 20 mg/kg) compared with the control, but the plasma concentrations of diltiazem coadministered with quercetin were not significant. The AUC and *C*_max⁡_ of the diltiazem in the rabbits pretreated with quercetin were significantly higher (*P* < 0.05, at 2 mg/kg; *P* < 0.01, at 10 and 20 mg/kg) than the control. The *AB* of diltiazem in the rabbits pretreated with quercetin was significantly (*P* < 0.05 at 2 mg/kg, *P* < 0.01 at 10 and 20 mg/kg) higher (9.10–12.81%) than the control (4.64%). The bioavailability of diltiazem in the rabbits pretreated with quercetin is increased significantly compared with the control, but not in the rabbits coadministered with quercetin [213].

#### 5.1.4. Quercetin and Tamoxifen

The pharmacokinetic parameters of tamoxifen in plasma were determined after oral administration of tamoxifen (10 mg/kg) with or without quercetin (2.5, 7.5 and 15 mg/kg). The coadministration of quercetin (2.5 and 7.5 mg/kg) significantly (*P* < 0.05) increased the *K*_*a*_, *C*_max⁡_, and AUC of tamoxifen. The *AB* of tamoxifen with 2.5 and 7.5 mg/kg quercetin ranged from 18.0 to 24.1%, which was significantly higher than the control group, 15.0% (*P* < 0.05). The *RB* of tamoxifen coadministered with quercetin was 1.20–1.61 times higher than the control group. The coadministration of quercetin caused no significant changes in the terminal *t*_1/2_ and *t*_max⁡_ of tamoxifen. The enhanced bioavailability of tamoxifen as a result of its coadministration with quercetin might be due to the effect of quercetin promoting the intestinal absorption and reducing the first-pass metabolism of tamoxifen [214].

#### 5.1.5. Quercetin and Fexofenadine

Short-term effect of quercetin on the pharmacokinetics of fexofenadine was studied in 12 healthy volunteers. Subjects were treated daily for 7 days with 500 mg quercetin or placebo 3 times a day. On day 7, a single dose of 60 mg fexofenadine was administered orally. Effects on AUC, *C*_max⁡_, *t*_1/2_, *K*_el_, and Cl_R_ are shown in Table 30 after administration of fexofenadine and in combination with quercetin. The results showed that short-term use of quercetin elevated the plasma concentrations of fexofenadine in humans [219].

#### 5.1.6. Quercetin and Etoposide

The effect of quercetin on the pharmacokinetics of etoposide was studied in rats. Etoposide was administered to rats orally (9 mg/kg) or intravenous (3 mg/kg) without or with quercetin (1, 5, or 15 mg/kg). The plasma concentration of etoposide was determined by HPLC with fluorescence detector. In the presence of quercetin, the pharmacokinetic parameters of etoposide were significantly altered in the oral group, but not in the intravenous group. The presence of quercetin significantly (5 mg/kg, *P* < 0.05; 15 mg/kg, *P* < 0.01) increased the AUC of orally administered etoposide from 43.0–53.2%. The presence of 5 or 15 mg/kg of quercetin significantly (*P* < 0.05) decreased the CL_*B*_ of oral etoposide. Consequently, compared to the control group (8.87%), the presence of quercetin significantly (5 mg/kg, *P* < 0.05; 15 mg/kg, *P* < 0.01) increased the *AB* of etoposide to 12.7 or 13.6%. The results from Table 31 indicated that oral bioavailability of etoposide was enhanced by quercetin [220].

#### 5.1.7. Quercetin and Epigallocatechin Gallate

Epigallocatechin gallate (EGCG) is a main anticancer component in green tea. The pharmacokinetic parameters after oral administration of green tea extract (GTE) and GTE + quercetin (Q). In rat, supplementations of GTE and GTE + Q raised the plasma *C*_max⁡_ were 55.29 ± 1.70 and 94.44 ± 1.59 ng/mL, respectively. The corresponding *t*_1/2_ elimination was 2.04 ± 0.2 h and 2.28 ± 0.049 h. The AUC_0−24 h_ was 510.16 ± 9.88 ng·h/mL and 794.08 ± 15.27 ng·h/mL (*P* ≤ 0.05), respectively. The results showed that quercetin increased bioavailability of EGCG in rats [221].

#### 5.1.8. Quercetin and Doxorubicin

The pharmacokinetic parameters of doxorubicin were determined in rats after oral (50 mg/kg) or intravenous (10 mg/kg) administration in the presence and absence of quercetin (0.6, 3 or 15 mg/kg). Compared to control, quercetin significantly increased the AUC and *C*_max⁡_ of oral doxorubicin, while there was no significant change in *t*_max⁡_ and *t*_1/2_ of doxorubicin. Consequently, the *AB* of doxorubicin was increased by quercetin compared to control, and the *RB* of oral doxorubicin was increased by 1.32–2.36-fold (Table 32). In contrast, the pharmacokinetics of intravenous doxorubicin was not affected by quercetin [215].

Park et al. [223] reported that quercetin did not affect the *in vivo* pharmacokinetics of intravenously administered doxorubicin.

### 5.2. Naringin

Naringin is the major flavonoid glycoside found in grapefruit, apples, onions, and tea [224]. Naringin exerts a variety of pharmacological effects such as antioxidant activity, antiulcer activity, antiallergic activity and anticancer activity [211], and blood lipid lowering. Naringin has been reported as a CYP3A4 inhibitor [225, 226] as well as a P-glycoprotein modulator [226, 227]. Park et al. [223] reported that naringin did not affect the *in vivo* pharmacokinetics of intravenously administered doxorubicin.

#### 5.2.1. Naringin and Diltiazem

Pharmacokinetic parameters of diltiazem were determined in rats following an oral administration of diltiazem (15 mg/kg) to rats in the presence and absence of naringin (5 and 15 mg/kg). Compared to the control given diltiazem alone, the *C*_max⁡_ and AUC of diltiazem increased by 2-fold in rats pretreated with naringin, while there was no significant change in *t*_max⁡_ and terminal *t*_1/2_ of diltiazem. Consequently, *AB* and *RB* values of diltiazem in the presence of naringin were significantly higher (*P* < 0.05) than those from the control group. Metabolite-parent AUC ratio in the presence of naringin decreased by 30% compared to the control group, implying that naringin could be effective to inhibit the metabolism of diltiazem. In conclusion, the concomitant use of naringin significantly enhanced the oral exposure of diltiazem in rats [228].

#### 5.2.2. Naringin and Paclitaxel

The effect of oral naringin on the pharmacokinetics of intravenous paclitaxel was studied in rats. Oral naringin (3.3 and 10 mg/kg) was pretreated 30 min before intravenous (3 mg/kg) administration of paclitaxel. After intravenous administration of paclitaxel, the AUC was significantly greater (40.8% and 49.1% for naringin doses of 3.3 and 10 mg/kg, resp.), and Cl_*B*_ was significantly slower (29.0% and 33.0% decrease, resp.) than controls. The significantly greater AUC could be due mainly to an inhibition of metabolism of paclitaxel via CYP3A1/2 by oral naringin. The inhibition of hepatic P-glycoprotein by oral naringin could also contribute to the significantly greater AUC of intravenous paclitaxel by oral naringin [229].

#### 5.2.3. Naringin and Verapamil

The effect of naringin on the pharmacokinetics of verapamil was studied in rabbits. The pharmacokinetic parameters of verapamil were determined after administering verapamil (9 mg/kg) orally to rabbits in the pretreated with naringin (1.5, 7.5, and 15 mg/kg). Naringin pretreatment significantly altered the pharmacokinetic parameters of verapamil. Compared with the control group (given verapamil alone), the *K*_*a*_, *C*_max⁡_, and AUC of verapamil were significantly increased in the pretreatment of naringin. However, there were no significant change in *t*_max⁡_ and *t*_1/2_ of verapamil. Consequently, pretreatment of naringin significantly increased the *AB* of verapamil significantly in a dose-dependent manner and elevated the *RB* of verapamil by 1.26–1.69-fold (Table 33). In conclusion, pretreatment of naringin enhanced the oral bioavailability of verapamil [222].

### 5.3. Capmul

Capmul is a glyceryl caprate produced from edible fats and oils. It is commonly used in lip products. Cho et al. [230] reported that capmul MCM C10 enhanced the bioavailability of ceftriaxone by 55–79% in rats.

### 5.4. Cow Urine Distillate

Cow urine distillate is more effective as bioenhancer than cow urine, to increase the effectiveness of antimicrobial, antifungal, and anticancer drugs [231].

Cow urine has antitoxic activity against the cadmium chloride toxicity and it can be used as a bioenhancer of zinc. Mature male mice exposed to cadmium chloride only showed 0% fertility rate. However, the animals exposed to cadmium chloride + cow urine distillate + zinc sulfate showed 90% fertility rate with 100% viability and lactation indices. Fertility index was also found to be 88% in group treated with cadmium chloride + cow urine distillate. Thus, these results indicate that cow urine distillate works as an antitoxic against the cadmium chloride toxicity and it can be used as a bioenhancer of zinc [232].

Cow urine distillate increased the activity of rifampicin by about 5–7 times against *Escherichia coli* and 3–11 times against Gram-positive bacteria. It probably acts by enhancing the transport of antibiotics across the membrane of gastrointestinal tract. The enhancement in transport is approximately 2–7 times [109].

Cow urine distillate enhanced the gonadotropin releasing hormone conjugate on the reproductive hormones and estrous cycle of female mice [233]. Cow urine distillate significantly enhanced the effect of gonadotropin releasing hormone on the gonadosomatic indices, sperm motility, sperm count, and sperm morphology, especially in 90- and 120-day-treated groups (*P* < 0.05) in male mice.Cow urine distillate enhanced these effects because of its immunomodulatory properties [234].

## 6. Conclusions

The effective formulation strategy for the optimization of the pharmacokinetic characteristics of dietary components is crucial to improve their *in vivo* performance and ultimately maximize their effectiveness as a bioavailability enhancer [235]. The available scientific research on bioenhancers has shown to produce significant enhancing effect on bioavailability when coadministered or pretreated with many drugs and nutraceuticals. These natural compounds include piperine, *Zingiber officinale*, niaziridin, glycyrrhizin, *Cuminum cyminum*,* Carum carvi*, allicin, lysergol, *Aloe vera*, *Stevia rebaudiana*, curcumin, sinomenine, genistein, *Ammannia multiflora*, capsaicin, quercetin, naringin, capmul and cow urine distillate. They reduce the dose, shorten treatment, and thus reduce drug-resistance and drug toxicity or adverse reactions. Due to dose economy, treatment is cost-effective. Bioenhancers are also found to decrease or having no effect or little effect on the bioavailability of some drugs [73, 128, 129].

The current paper discussed the enhancing effects of bioenhancers of drugs in animals and humans but these compounds have not been completely explored in experimental animals till date. However, these studies lack information on their exact mechanism of action, toxicity evaluation of extracts, and suitable combinations. Therefore, we have to focus on this area for further research on their active principles, mechanisms of actions, toxicity evaluation, and suitable combinations with other drugs. So we can explore novel principles with high bioenhancing ability and less toxic effects. 

## Figures and Tables

**Table 1:** Mechanisms of action of herbal bioenhancers.

Mechanisms of action	References
Bioenergetic properties	Reanmongkol et al. [9], Jamwal and Singh [10]
Increases gastrointestinal blood supply and reduces hydrochloric acid secretion	Annamalai and Manavalan [11]
Stimulation of *γ*-glutamyl transpeptidase (GGT) activity which enhances uptake of amino acids	Johri et al. [12]
Cholagogues effect	Majeed et al. [13]
Thermogenic and bioenergetics properties	Majeed et al. [13]
Inhibition of gastric emptying time, gastrointestinal transit	Bajad et al. [14]
Inhibition of drug metabolizing enzymes and suppression of first pass metabolism	Atal et al. [15], Reen et al. [16], and Bhardwaj et al. [17]
Modifications in GIT epithelial cell membrane permeability	Khajuria et al. [18]

**Table 2:** Drug metabolizing enzymes inhibited by piperine.

Drug metabolizing enzymes	References
Arylhydrocarbon hydroxylase (AHH)	Atal et al. [15], Singh et al. [66]
Uridine diphosphate- (UDP-) glucuronyl transferase	Atal et al. [15], Singh et al. [66]
Ethylmorphine-N-demethylase	Atal et al. [15], Singh et al. [66]
7-Ethoxycoumarin-O-deethylase	Atal et al. [15], Singh et al. [66]
3-Hydroxy-benzo(a)pyrene glucuronidation	Atal et al. [15], Singh et al. [66]
UDP-glucose dehydrogenase (UDP-GDH)	Reen et al. [16]
5-lipoxygenase	Stöhret al. [67]
Cyclooxygenase-1	Stöhret al. [67]
Cytochrome P450	Atal et al. [15], Singh et al. [66], Bhardwaj et al. [17]

**Table 3:** Drugs bioenhanced by piperine.

Drugs	Experimental model	References
Vasicine	Rats	Zutshi and Kaul [74]
Pyrazinamide	*In vitro*	Zutshi [75]
Phenytoin, propranolol, theophyllin, spartein, sulphadiazine, and tetracycline	Human volunteers	Bano et al. [76, 77]
Pentobarbitone	Human volunteers	Mujumdar et al. [78]
Curcumin	Human volunteers and rats	Shoba et al. [79]
	*In vivo*	Shaikh et al. [80]
	Rats	Suresh and Srinivasan [81]
Nimesulide	Mice	Gupta et al. [82]
Indomethacin	Rabbits	Karan et al. [83]
Oxyphenylbutazone	Rats	Mujumdar et al. [84]
Phenytoin	Human volunteers	Velpandian et al. [85], Pattanaik et al. [86]
Rifampicin	Human	Zutshi et al. [87]
	*In vitro*	Balakrishnan et al. [88]
Amoxycillin trihydrate and cefotaxime	Rats	Hiwale et al. [89]
EGCG [(−)-epigallocatechin-3-gallate]	Mice	Lambert et al. [90]
Oxytetracycline	WLH hens	Singh et al. [91]
Ciprofloxacin	Rabbits	Balkrishna and Yogesh [92]
	*In vitro*	Khan et al. [8]
Nevirapine	Human	Kasibhatta and Naidu [93]
Diclofenac sodium and pentazocine	Albino mice	Pooja et al. [47]
Pefloxacin	Gaddi goats	Dama et al. [94]
Ampcilllin and norloxacin	Rabbits	Janakiraman and Manavalan [95]
Carbamazepine	*In vitro*	Pattanaik et al. [96]
Fexofenadine	Rats	Jin and Han [97]
Metronidazole	Rabbits	Singh et al. [98]
Ampicillin trihydrate	Human	Janakiraman and Manavalan [99]
Resveratrol	Mice	Johnson et al. [100]
Gatifloxacin	Layer birds	Patel et al. [101]
	Broiler birds	Devada et al. [102]
Atenolol	Rats	Singh and Chand [103]
Ibuprofen	*In vitro*	Venkatesh et al. [104]
Losartan potassium	Rats	Singh et al. [105]

**Table 4:** Nutraceuticals bioenhanced by piperine [13, 19].

Class	Examples
Water soluble vitamins	Vitamin B_1_, Vitamin B_2_, niacinamide, Vitamin B_6_, Vitamin B_12_, folic acid, and Vitamin C
Fat soluble vitamins	Vitamin A, *β*-carotene (provitamin), Vitamin D, Vitamin E, and Vitamin K
Amino acids	Lysine, isoleucine, leucine, threonine, valine, tryptophan, phenylalanine, and methionine
Minerals	Iodine, calcium, iron, zinc, copper, selenium, magnesium, potassium, and manganese
Herbal compounds	Boswellic acid (*Boswellia serrata*), Ginsenosides (*Gingko biloba*), Withanaloids (*Withania somnifera*), Curcuminoides (*Curcuma longa*), and Pycnogenol (*Pinus pinaster*)

**Table 5:** Comparative pharmacokinetic parameters after oral supplementation of curcumin and curcumin + piperine in human volunteers [79].

Parameters	Curcumin	Curcumin (2** **g/kg) + piperine
	(2** **g/kg)	(20** **mg/kg)
*C*_max⁡_ (*μ*g/mL)	1.35 ± 0.23	1.80 ± 0.16
*t*_max⁡_ (h)	0.83 ± 0.05	1.29 ± 0.23
*t*_1/2_ (h)	1.70 ± 0.58	1.05 ± 0.18
AUC (*μ*g·h/mL)	2.36 ± 0.28	3.64 ± 0.31
Cl_*B*_ (L/h)	713 ± 12	495.90 ± 193.90

**Table 6:** Effect of piperine on the serum *β*-carotene levels during a 14-day supplementation in human volunteers [20].

Treatment (*n* = 6)	*β*-carotene (*μ*g/dL)			AUC (*μ*g/dL) × (time in days)
	Day 0	Day 14	Change
*β*-carotene + placebo	16.3 ± 2.6	47.2 ± 6.4	30.9 ± 5.4	272 ± 47.6
*β*-carotene + piperine	16.0 ± 3.1	65.8 ± 9.7	49.8 ± 9.6	435 ± 74.2

**Table 7:** Effect of piperine on pharmacokinetics of rifampicin, isoniazid, and pyrazinamide combination in human volunteers [107, 108].

Kinetic parameters	Formulation A			Formulation B		
	Rifampicin	Isoniazid	Pyrazinamide	Rifampicin**	Isoniazid**	Pyrazinamide**
*t*_1/2_ (h)	0.67 ± 0.008	0.64 ± 0.01	0.60 ± 0.03	0.40 ± 0.04	0.42 ± 0.04	0.37 ± 0.008
*t*_1/2(el)_ (h)	8.40 ± 0.06	7.90 ± 0.10	9.50 ± 0.14	11.1 ± 0.18	8.15 ± 0.26*	11.0 ± 0.17
*C*_max _ (*μ*g/mL)	8.20 ± 0.46	2.56 ± 0.17	27.0 ± 2.0	17.6 ± 1.09	10.07 ± 0.90	40.0 ± 1.64
AUC_0–∞_ (*μ*g·h/mL)	104.6 ± 4.38	41.0 ± 1.72	318.6 ± 8.05	143.95 ± 5.85	94.95 ± 3.98	435.41 ± 9.7

*Not significant (*P* > 0.05), **highly significant (*P* < 0.001).

Formulation A: rifampicin—450 mg, isoniazid—300 mg, pyrazinamide—1500 mg.

Formulation B: rifampicin—450 mg, isoniazid-300 mg, pyrazinamide—1500 mg, piperine—20 mg.

**Table 8:** Comparison of mean plasma levels of oxytetracycline (*μ*g/mL) at different time intervals following oral administration in control and *P. longum-*treated WLH birds [91].

Time (Min)	Control	Treated
15	0.23 ± 0.024	0.30 ± 0.022*
30	0.34 ± 0.025	0.45 ± 0.029**
60	0.72 ± 0.051	0.77 ± 0.046
120	0.58 ± 0.037	0.63 ± 0.030
240	0.37 ± 0.015	0.47 ± 0.034**
360	0.26 ± 0.017	0.38 ± 0.021****
480	0.23 ± 0.015	0.31 ± 0.019***
600	0.19 ± 0.005	0.25 ± 0.015***

*n* = 8, mean ± SE; *P < 0.1, **P < 0.05, ***P < 0.01, ****P < 0.001.

**Table 9:** Comparative pharmacokinetic parameters of oxytetracycline administered orally (10 mg/kg) in control and *P. longum* treated birds [91].

Parameters	Control OTC	Treated OTC + piperine
*β* (/h)	0.147 ± 0.011	0.112 ± 0.006*
*t*_1/2β_ (h)	4.934 ± 0.422	6.370 ± 0.438**
AUC (*μ*g·h/mL)	5.055 ± 0.689	6.417 ± 0.317*
AUMC (*μ*g·h^2^/mL)	43.62 ± 11.15	63.58 ± 7.042*
MRT (h)	7.98 ± 0.744	9.77 ± 0.644**
*t*_*d*_ (h)	16.38 ± 1.4	21.142 ± 1.453**
Cl_*B*_ (mL/kg/h)	184.63 ± 7.7	145.97 ± 14.42**

*n* = 8, mean ± SE; *P < 0.01, **P < 0.05.

**Table 10:** MIC of ciprofloxacin for *S. aureus* ATCC 29213 at different concentrations of piperine [8].

Piperine conc.	MIC (*μ*g) of ciprofloxacin for *S. aureus *
(*μ*g/mL)	ATCC 29213
0.0	0.25
12.5	0.12
25	0.12
50	0.06

**Table 11:** Drug susceptibilities of *S. aureus *ATCC 29213 and the ciprofloxacin selected mutant (CIP^r^-1) [8].

Compound	MIC (*μ*g/mL)	
	*S. aureus *ATCC 29213	CIP^r^-1
Ethidium bromide	4	8
Ethidium bromide + piperine	1	2
Ciprofloxacin	0.25	128
Ciprofloxacin + piperine	0.12	8

**Table 12:** Effect of piperine on plasma concentration of ampicillin in rabbits [95].

Time (h)	Ampicillin (***μ***g/mL)	Ampicillin + piperine (***μ***g/mL)
0.0	0	0
0.5	30.584 ± 0.38	35.481 ± 0.26
1.0	44.668 ± 0.32	251.188 ± 0.27
1.5	31.672 ± 0.49	188.364 ± 0.31
2.0	26.607 ± 0.37	84.139 ± 0.31
2.5	18.836 ± 0.38	66.834 ± 0.26
3.0	17.782 ± 0.32	35.481 ± 0.27
3.5	12.589 ± 0.31	33.496 ± 0.26
4.0	12.589 ± 0.32	26.607 ± 0.27
4.5	9.441 ± 0.26	26.607 ± 0.27

*n* = 6, mean ± SD, *P* < 0.05.

**Table 13:** Effect of piperine on plasma concentration of norfloxacin in rabbits [95].

Time (h)	Norfloxacin (*μ*g/mL)	Norfloxacin + piperine (*μ*g/mL)
0.0	0	0
1.0	10.593 ± 0.26	14.125 ± 0.27
2.0	7.079 ± 0.26	17.783 ± 0.32
3.0	5.623 ± 0.32	14.963 ± 0.27
4.0	5.011 ± 0.38	11.885 ± 0.32
5.0	4.732 ± 0.26	10.592 ± 0.37
6.0	3.981 ± 0.26	8.414 ± 0.25
7.0	3.548 ± 0.21	8.414 ± 0.26
8.0	3.349 ± 0.26	7.079 ± 0.26
9.0	3.162 ± 0.27	7.079 ± 0.27
10.0	2.512 ± 0.27	5.623 ± 0.27
11.0	2.238 ± 0.26	5.011 ± 0.27
12.0	1.778 ± 0.26	4.467 ± 0.27

*n* = 6, mean ± SD, *P* < 0.

**Table 14:** Pharmacokinetic parameters of ampicillin, norfloxacin, and in combination with piperine after oral treatment in rabbit [95].

Treatment	*t*_max⁡_ (h)	*C*_max⁡_ (*μ*g/mL)	*t*_1/2_ (h)	AUC (*μ*g·h/mL)
Ampicillin (150** **mg/kg)	1 ± 0.31	44.6 ± 0.27	1.3 ± 0.46	103.7 ± 0.52
Ampicillin (150** **mg/kg) + piperine (20** **mg/kg)	1.1 ± 0.3	251.2 ± 0.28	1.9 ± 0.57	350.49 ± 0.47
Norfloxacin (150** **mg/kg)	3.1 ± 0.32	11 ± 0.26	1.75 ± 0.38	63.98 ± 0.51
Norfloxacin (150** **mg/kg) + piperine (20** **mg/kg)	3.2 ± 0.31	16.1 ± 0.27	2.97 ± 0.38	111.69 ± 0.54

*n* = 6, mean ± SD, *P* < 0.05.

**Table 15:** Comparative pharmacokinetic parameters after oral administration of metronidazole and in combination with piperine in rabbits [98].

Pharmacokinetic	Metronidazole	Metronidazole + piperine
parameters		
*C*_max⁡_ (ng/mL)	3,805.89 ± 233.8	6,007.07 ± 348.8
AUC_0–24_ (ng·h/mL)	45,073.75 ± 713.7	84,980.98 ± 345.6
*t*_max⁡_ (h)	1.66 ± 0.57	1.66 ± 0.57
*K*_el_ (mL/h)	0.06 ± 0.02	0.04 ± 0.02
*t*_1/2_ (h)	1.48 ± 1.23	12.24 ± 1.04
*V*_*d*_ (L)	2.69 ± 0.23	1.48 ± 0.65

**Table 16:** Comparison of plasma concentrations (*μ*g/mL) of gatifloxacin- and gatifloxacin + piperine-treated birds after single oral administration (10 mg/kg) [101].

Time (h)	Gatifloxacin	Gatifloxacin + piperine
0.5	0.48 ± 0.013	0.53 ± 0.009**
0.75	1 ± 0.014	1.16 ± 0.029**
1	1.35 ± 0.016	1.46 ± 0.021*
2	1.74 ± 0.023	2.14 ± 0.020
4	1.35 ± 0.02	1.47 ± 0.034*
8	0.97 ± 0.009	1.08 ± 0.019**
12	0.46 ± 0.01	0.54 ± 0.008

*n* = 6, *Significant at *P* < 0.05, **Highly significant at *P* < 0.01.

**Table 17:** Comparison of pharmacokinetic parameters of gatifloxacin- and gatifloxacin + piperine-treated layer birds after single oral administration (10 mg/kg) [101].

Pharmacokinetic	Gatifloxacin	Gatifloxacin + piperine
parameters		
*C*_max⁡_ (*μ*g/mL)	1.74 ± 0.023	2.14 ± 0.019*
AUC (*μ*g·h/mL)	15.25 ± 0.219	17.54 ± 0.204*
*t*_max⁡_ (h)	2 ± 0.0	2 ± 0.0
*t*_1/2_ (h)	3.74 ± 0.073	4.03 ± 0.097*
*V*_*d*_ (L/kg)	3.54 ± 0.038	3.33 ± 0.083*
*F* (%)	74.52 ± 1.021	85.74 ± 0.956*

*n* = 6, mean ± SE; **Highly significant at *P* < 0.01, *Significant at *P* < 0.05.

**Table 18:** Comparison of pharmacokinetic parameters of atenolol- and atenolol + piperine-treated male New Zealand white rats after single oral administration (100 mg/kg) [103].

Pharmacokinetic	Atenolol	Atenolol + piperine
parameters		
*C*_max⁡_ (pg/mL)	22,453 ± 233	37,860 ± 348
AUC_0–24_ (pg·h/mL)	1,07,189 ± 1615	1,52,011 ± 2354
*t*_max⁡_ (h)	2.0 ± 0.0	2.0 ± 0.0
*K*_el_ (mL/h)	0.27 ± 0.005	0.25 ± 0.010
*t*_1/2_ (h)	6.27 ± 0.057	6.72 ± 0.100
*V*_*d*_ (L)	4.54 ± 0.786	2.88 ± 0.305

*n* = 3, mean ± SE.

**Table 19:** Comparison of pharmacokinetic parameters of losartan potassium- and losartan potassium + piperine-treated male New Zealand white rats after single oral administration (100 mg/kg) [105].

Pharmacokinetic	Losartan potassium	Losartan potassium
parameters		+ piperine
*C*_max⁡_ (pg/mL)	32,461 ± 1436	48,399 ± 6060
AUC_0–24_ (pg·h/mL)	1,10,441 ± 5280	1,69,924 ± 14,343
*t*_max⁡_ (h)	1.0 ± 0.0	1.0 ± 0.0
*K*_el_ (mL/h)	0.295 ± 0.002	0.283 ± 0.022
*t*_1/2_ (h)	2.34 ± 0.01	2.44 ± 0.195
*V*_*d*_ (L)	3.0 ± 0.15	2.0 ± 0.25

*n* = 3, mean ± SE.

**Table 20:** Percent enhancement in bioavailability of different drugs/compounds by supplementation of bioactive fractions from *Z. officinale *and its combination with piperine [128, 129].

Category	Compound	Percent enhancement	
		BE from *Z. officinale *	BE from *Z. officinale* + piperine
Macrolides	Azithromycin	78	85
	Erythromycin	68	105
	Roxithromycin	72	93
Cephalosporins	Cefalexin	75	85
	Cefadroxil	68	65
Penicillins	Amoxycillin	80	90
	Cloxacillin	76	90
Aminoglycosides	Kanamycin	65	92
Fluoroquinolones	Ciprofloxacin	68	70
	Pefloxacin	53	69
Antifungal	Fluconazole	120	110
	Ketoconazole	125	100
Antiviral	Acyclovir	82	85
	Zidovudine	105	126
CNS drugs	Alprazolam	76	70
Anticancer	Methotrexate	87	49
	5-Fluorouracil	110	93
	Doxorubicin	72	75
	Cisplatin	56	45
Cardiovascular	Amlodipine	68	95
	Propranolol	76	104
	Lisinopril	76	100
Anti-inflammatory/Antiarthritic	Diclofenac	90	140
	Nimesulide	144	165
	Piroxicam	86	134
Antituberculosis/Antileprosy	Rifampicin	65	98
	Dapsone	46	55
	Ethionamide	56	48
	Cycloserine	71	70
Antihistamines	Salbutamol	78	92
	Theophylline	76	80
	Bromhexine	46	75
Corticosteroids	Dexamethasone	76	80
	Betamethasone	75	70
Immunosuppressants	Cyclosporin A	116	120
	Tacrolimus	75	117
Antiulcer	Ranitidine	147	165
	Cimetidine	98	76
Vitamins	Vitamin A (1 mg/kg)	30	40
	Vitamin E (40 mg/kg)	27	25
	Vitamin C (50 mg/kg)	25	24
	Folic acid (50 *μ*g/kg)	34	35
Antioxidants	*β*-Carotene (15 mg/kg)	36	55
	Silymarin (5 mg/kg)	28	43
Herbal products	Curcumin (50 mg/kg)	43	70
	Rutin (40 mg/kg)	21	78
Herbal plants	*Echinacea *	66	82
	*Tinospora cordifolia *	67	112
	*Picrorhiza kurroa*	56	87
	*Andrographis paniculata*	55	70
	*Emblica ribes*	65	72
	*Asparagus racemosus*	44	60
	*Withania somnifera*	64	48
Amino acids	Methionine (20 mg/kg)	25	45
	Lysine (40 mg/kg)	15	40
	Leucine (50 mg/kg)	17	34
	Valine (25 mg/kg)	25	46
	Isoleucine (25 mg/kg)	31	35
Essential elements	Zinc (0.1 mg/kg)	19	20
	Potassium (25 mg/kg)	21	19

**Table 21:** Enhancement of antibiotic activity by niaziridin* in combination with antibiotics against different organisms [145, 146].

Organisms	Antibiotics	Concentration (*μ*g/mL)	Fold enhancement of activity
*E. coli* (CA8000)	Rifampicin	20.0	38.8
	Rifampicin	30.0	12.6
	Ampicillin	6.0	5.3
	Tetracycline	1.0	2.3
	Tetracycline	2.0	5.2
	Nalidixic acid	6.0	50.0
*B. subtilis* (MTCC121)	Rifampicin	0.05	19.4
	Ampicillin	0.01	5.1
	Tetracycline	1.0	3.5
	Nalidixic acid	2.0	7.0
*M. smegmatis* (MC2 155)	Rifampicin	0.05	1.9
	Ampicillin	0.01	4.7
	Tetracycline	1.0	4.0
	Nalidixic acid	2.0	1.2

*Dose: *E. coli* = 0.1 *μ*g/kg; *B. subtilis *and *M. smegmatis *= 1.0 *μ*g/kg.

**Table 22:** Enhancement of antibiotic activity by glycyrrhizin (1 *μ*g/mL) in combination with antibiotics against different organisms [154, 155].

Organisms	Antibiotics	Concentration (*μ*g/mL)	Fold enhancement of activity
*E. coli* (ATCC 10536)	Rifampicin	10.0	3.8
	Rifampicin	20.0	14.0
	Nalidixic acid	8.0	9.5
	Ampicillin	6.0	12.6
	Ampicillin	8.0	2.1
	Tetracycline	2.0	1.9
	Tetracycline	4.0	9.0
*B. subtilis* (ATCC 6015)	Nalidixic acid	6.0	4.5
	Nalidixic acid	8.0	6.75
	Tetracycline	1.0	5.0
*M. smegmatis* (ATCC 14468)	Rifampicin	0.5	6.5
	Nalidixic acid	4.0	7.7
	Tetracycline	0.5	1.5

**Table 23:** Percent enhancement in bioavailability of different compounds by supplementation of bioactive fractions from *C. cyminum* and its combination with piperine [166, 167].

Category	Compound	Percent enhancement	
		BE from *C. cyminum *	BE from *C. cyminum* + piperine
Macrolides	Azithromycin	83	97
	Erythromycin	75	95
	Roxithromycin	67	103
Cephalosporins	Cefalexin	60	75
	Cefadroxil	90	79
Penicillins	Amoxycillin	75	98
	Cloxacillin	94	95
Aminoglycosides	Kanamycin	95	110
Fluoroquinolones	Ciprofloxacin	52	47
	Pefloxacin	47	57
	Ofloxacin	61	73
Antifungal	Fluconazole	170	126
	Ketoconazole	136	156
Antiviral	Acyclovir	110	98
	Zidovudine	330	415
CNS drugs	Alprazolam	60	104
Anticancer	Methotrexate	125	30
	5-Fluorouracil	335	435
	Doxorubicin	85	103
	Cisplatin	70	79
Cardiovascular	Amlodipine	55	103
	Propranolol	135	210
	Lisinopril	83	98
Anti-inflammatory/Antiarthritic	Diclofenac	65	108
	Nimesulide	168	150
	Piroxicam	70	107
Antituberculosis/Antileprosy	Rifampicin	250	365
	Dapsone	60	75
	Ethionamide	78	65
	Cycloserine	89	90
Antihistamines	Salbutamol	110	85
	Theophylline	87	75
	Bromhexine	50	90
Corticosteroids	Dexamethasone	85	105
	Betamethasone	95	82
Immuno-suppressants	Cyclosporin A	156	275
	Tacrolimus	75	117
Antiulcer	Ranitidine	117	89
	Cimetidine	123	105
Vitamins	Vitamin A (1 mg/kg)	26	18
	Vitamin B_1_ (10 mg/kg)	37	33
Antioxidants	*β*-Carotene (15 mg/kg)	45	53
	Silymarin (5 mg/kg)	32	41
Herbal products	Curcumin (50 mg/kg)	39	29
Herbal plants	*Echinacea *	72	90
	*Tinospora cordifolia *	98	152
	*Picrorhiza kurroa*	78	115
	*Andrographis paniculata*	72	68
	*Emblica ribes*	72	60
	*Asparagus racemosus*	35	72
	*Withania somnifera*	55	65
Amino acids	Methionine (20 mg/kg)	27	30
	Lysine (40 mg/kg)	35	29
	Leucine (50 mg/kg)	31	32
	Valine (25 mg/kg)	25	24
	Isoleucine (25 mg/kg)	40	22
Essential elements	Iron (0.5 mg/kg)	23	29

**Table 24:** Percent enhancement in bioavailability of different drugs/compounds by supplementation of bioactive fractions from *C. carvi* and its combination with piperine and *Z. officinale *[5, 175].

Category	Compound	Dose (mg/kg)	Percent enhancement		
			BE from *C. carvi *	*C. carvi *+ piperine	*C. carvi *+ *Z. officinale *
Macrolides	Azithromycin	25	55	90	86
	Erythromycin	45	70	100	105
	Roxithromycin	15	65	95	98
Cephalosporins	Cefalexin	45	Nil	90	79
	Cefadroxil	45	67	95	85
	Ceftrioxone	25	72	78	75
	Cefixime	40	80	79	82
	Cefidinir	40	89	95	130
Penicillins	Amoxicillin	45	75	115	100
	Cloxacillin	25	110	95	110
Aminoglycosides	Amikacin	50	85	100	92
	Kanamycin	50	Nil	87	68
Fluoroquinolones	Ciprofloxacin	45	78	110	133
	Pefloxacin	40	Nil	70	75
	Ofloxacin	20	65	167	170
	Norfloxacin	40	55	65	60
Antifungal	Fluconazole	65	65	98	110
	Amphotericin B	78	78	90	80
	Ketoconazole	55	55	100	96
Antiviral	Acyclovir	40	78	100	90
	Zidovudine	10	92	95	87
CNS drugs	Alprazolam	0.1	Nil	70	80
	Haloperidol	0.5	95	90	85
Anticancer	Methotrexate	5	76	89	102
	5-Fluorouracil	25	90	110	100
	Doxorubicin	5	Nil	70	69
	Cisplatin	5	Nil	Nil	55
Cardiovascular	Amlodipine	1	Nil	50	65
	Lisinopril	1	79	95	90
	Atenolol	5	100	93	97
	Propranolol	8	68	90	75
Anti-inflammatory/antiarthritic	Diclofenac	5	Nil	100	95
	Piroxicam	2	Nil	98	76
	Nimesulide	10	100	140	145
	Rofecoxib	2.5	75	70	80
Antituberculosis/antileprosy	Rifampicin	40	110	170	140
	Pyrazinamide	12.5	45	50	55
	Dapsone	10	56	67	68
	Ethionamide	25	68	65	70
	Cycloserine	40	70	80	75
Antihistamines	Salbutamol	0.8	75	89	80
	Theophylline	30	70	79	89
	Bromhexine	25	Nil	70	71
	Loratidine	1	76	70	80
Corticosteroids	Prednisolone	4	65	67	60
	Dexamethasone	0.05	72	77	73
	Betamethasone	0.1	80	89	77
Immuno-suppressants	Cyclosporin A	10	100	105	120
	Tacrolimus	5	90	95	114
Antiulcer	Ranitidine	30	67	70	150
	Cimetidine	40	72	84	100
	Omeprazole	2	76	70	75
Vitamins	Vitamin A	1	19	16	27
	Vitamin B_1_	10	42	26	55
Antioxidants	*β*-Carotene	15	55	59	72
	Silymarin	5	38	45	41
Herbal products	Curcumin	50	48	51	52
	Rutin	40	45	40	42
Herbal plants	*Echinacea agustifolia*	10	Nil	76	65
	*Tinospora cordifolia *	50	76	90	71
	*Picrorhiza kurroa*	50	80	110	76
	*Aegles marmelos*	1000	65	65	60
	*Andrographis paniculata*	50	68	72	54
	*Asparagus racemosus*	50	Nil	55	45
	*Terminalia chebula*	50	92	87	91
	*Withania somnifera*	60	76	70	76
	*Centella asiatica *	30	68	65	62
Amino acids	Methionine	20	28	30	37
	Lysine	40	29	38	43
	Leucine	50	21	32	40
	Valine	25	19	29	38
	Isoleucine	25	34	44	50

**Table 25:** Enhancement of fungicidal activity of AmB by Allicin against *S. cerevisiae* [187].

Drug/compound	Conc. (*μ*g)	Action against *S. cerevisiae *
AmB alone	1	Mostly resistance to action
AmB alone	5	Subjected to lethal damage
Allicin alone	120	Not lethal
AmB + allicin	0.5 + 120	More susceptible to fungicidal activity of AmB

**Table 26:** Enhancement of antibiotic activity by lysergol (10 *μ*g/mL) in combination with rifampicin against different organisms [2].

Organisms	Antibiotics	Concentration (*μ*g/mL)	Fold enhancement of activity
*E. coli* (ATCC 10536)	Rifampicin	10	6–12
	Rifampicin	20	3–5
*B. subtilis *(ATCC 6051)	Rifampicin	0.4	3–4.6
*M. smegmatis* (ATCC 14468)	Rifampicin	0.2	4.5–6

**Table 27:** AUC of vitamins C and E alone or with Aloe gel or Aloe whole leaf extract [196].

Supplement	AUC (*μ*Mh)
Vitamin C (500 mg)	339 ± 124
Vitamin C + Aloe whole leaf extract	272 ±144
Vitamin C + Aloe gel	1031 ± 513
Vitamin E (420 mg)	19.3 ± 23.2
Vitamin E + Aloe whole leaf extract	38.3 ± 17.0
Vitamin E + Aloe gel	71.3 ± 22.5

mean ± SE.

**Table 28:** Pharmacokinetic parameters of paeoniflorin (150 mg/kg) and in combination with sinomenine (90 mg/kg) after oral administration in rats [201].

Parameters	Paeoniflorin	Sinomenine + paeoniflorin
AUC_0–∞_ (*μ*g·h/mL)	124.62 ± 36.91	1540.43 ± 548.96**
*C*_max⁡_ (*μ*g/mL)	1.26 ± 0.23	6.03 ± 2.45*
*t*_max⁡_ (min)	45.00 ± 5.00	77.30 ± 17.50*
*t*_1/2_ (min)	19.58 ± 9.01	53.78 ± 22.17***
Cl_*B*_ (mL/kg/min)	1301.83 ± 429.03	110.44 ± 45.61*
*V*_*d*_ (mL/kg)	102044.14 ± 46608.43	16064.25 ± 17189.33***
MRT (min)	133.12 ± 38.63	224.07 ± 26.62*

*n* = 6, mean ± SD; *P < 0.01, **P < 0.001, ***P < 0.05.

**Table 29:** Pharmacokinetic parameters of paclitaxel (40 mg/kg) and in combination with pretreated quercetin rats after oral administration [218].

Parameters	Paclitaxel control	Quercetin pretreatment				
		2 mg/kg	10 mg/kg	20 mg/kg	3 days, 10 mg/kg	3 days, 20 mg/kg
AUC (ng·h/mL)	1605 ± 409	2835 ± 721*	4205 ± 1103**	4978 ± 1211**	5123 ± 1263**	5301 ± 1328**
*C*_max⁡_ (ng/mL)	104 ± 27	161 ± 31*	248 ± 63**	279 ± 71**	288 ± 74**	299 ± 76**
*t*_max⁡_ (h)	2.0 ± 0.6	1.6 ± 0.5	1.5 ± 0.4	1.5 ± 0.5	1.5 ± 0.4	1.5 ± 0.4
*t*_1/2_ (h)	9.9 ± 2.5	13.9 ± 3.5	16.6 ± 4.4*	17.5 ± 4.5*	17.6 ± 4.6*	17.9 ± 4.7*
MRT (h)	15 ± 3.8	22 ± 5.6	25 ± 6.4*	26 ± 6.5*	26 ± 6.4*	26 ± 6.5*
*AB* (%)	2.0	3.5*	5.3**	6.2**	6.4**	6.6**
*RB* (%)	100	176	261	309	318	329

*n* = 8, mean ± SD; *P < 0.05; **P < 0.01, significant difference compared to control.

**Table 30:** Pharmacokinetic parameters of fexofenadine (60 mg) and in combination quercetin (500) mg after a single-dose administration [50].

Parameters	Fexofenadine	Fexofenadine + quercetin
AUC_0–∞_ (ng·h/mL)	2,075.5 ± 461.7	3,227.1 ± 665.9
*C*_max⁡_ (ng/mL)	295.3 ± 135.4	480.3 ± 163.7
*t*_max⁡_ (h)	2.0 (0.5–5)	2 (1.5–3)
*K*_el_ (/h)	0.16 ± 0.02	0.16 ± 0.05
*t*_1/2_ (h)	4.5 ± 0.8	4.7 ± 1.5
Cl_*R*_ (L/h)	4.72 ± 1.13	4.29 ± 1.40

Data are expressed as mean ± SD except for *t*_max⁡_, which is expressed as median (range).

**Table 31:** Pharmacokinetic parameters of etoposide (9 mg/kg) and in combination with quercetin after oral administration in rats [220].

Parameters	Etoposide control	Quercetin + etoposide		
		1 mg/kg	5 mg/kg	15 mg/kg
AUC (ng·h/mL)	1226 ± 154	1419 ± 222	1753 ± 315*	1878 ± 364**
*C*_max⁡_ (ng/mL)	484 ± 74.7	455 ± 80.5	586 ± 89.5*	673 ± 93.3*
Cl_*B*_ (mL/kg/h)	8265 ± 1566	7178 ± 1391	5980 ± 1292*	5481 ± 1167*
*t*_max⁡_ (h)	0.500 ± 0.158	0.667 ± 0.129	0.693 ± 0.154	0.708 ± 0.188
*K*_el_ (/h)	0.255 ± 0.031	0.230 ± 0.027	0.225 ± 0.025	0.221 ± 0.018
*t*_1/2_ (h)	2.75 ± 0.32	3.05 ± 0.37	3.11 ± 0.39	3.16 ± 0.41
*AB* (%)	8.87 ± 1.33	10.3 ± 1.55	12.7 ± 1.96*	13.6 ± 2.24**
*RB* (%)	100	116	143	153

*n* = 6, mean ± SD; *P < 0.05, **P < 0.01 compared to control.

**Table 32:** Pharmacokinetic parameters of doxorubicin (50 mg/kg) and in the presence or absence (control) of quercetin (0.6, 3, and 15 mg/kg) after oral administration [215].

Parameters	Doxorubicin (control)	Doxorubicin + quercetin		
		0.6 mg/kg	3 mg/kg	15 mg/kg
AUC_0–∞_ (ng·h/mL)	186 ± 44.6	244 ± 63.2*	344 ± 98.6**	439 ± 107.3**
*C*_max⁡_ (ng/mL)	20.2 ± 5.13	27.3 ± 6.42*	38.0 ± 9.28*	45.6 ± 11.16**
*t*_max⁡_ (h)	0.25	0.25	0.25	0.25
*V*_ss_ (L/min)	6.54 ± 1.45	5.06 ± 1.13	2.88 ± 0.64*	2.23 ± 0.52*
*t*_1/2_ (h)	13.5 ± 3.32	13.7 ± 3.41	13.8 ± 3.51	13.9 ± 3.82
*AB* (%)	3.04 ± 0.74	4.01 ± 0.94*	5.58 ± 1.32**	7.12 ± 1.71**
*RB* (%)	100	132	185	236

*n* = 7, mean ± SD; *P < 0.05, **P < 0.01 significant difference compared to control.

**Table 33:** Pharmacokinetic parameters of verapamil (9 mg/kg) and in combination with naringin pretreated rabbits after oral administration [222].

Parameters	Verapamil control	Naringin pretreatment		
		1.5 mg/kg	7.5 mg/kg	15 mg/kg
AUC (ng·h/mL)	291 ± 75.7	370 ± 96.1*	445 ± 1025**	494 ± 142**
*C*_max _ (ng/mL)	55.3 ± 14.4	66.5 ± 17.3	86.9 ± 53.3**	92.6 ± 54.5**
*t*_max⁡_ (h)	0.25 ± 0.07	0.25 ± 0.08	0.25 ± 0.06	0.25 ± 0.08
*K*_*a*_(/h)	3.8 ± 0.99	5.1 ± 1.30	6.1 ± 1.59*	6.8 ± 1.72*
*t*_1/2_ (h)	12.0 ± 3.12	13.4 ± 3.46	13.6 ± 3.51	13.9 ± 3.61
*AB* (%)	8.8 ± 2.3	11.2 ± 2.8*	13.5 ± 3.2**	15.0 ± 3.4**
*RB* (%)	100	126	153	169

*n* = 6, mean ± SD; *P < 0.05, **P < 0.01, significant difference compared to control.

## Abbreviations

AB: Absolute bioavailability
AUC: Area under the plasma concentration-time curve
AUC: Area under the plasma concentration-time curve from 0 h to infinity
AUC: Area under the plasma concentration-time curve from 0 h to 24 h
AUC_*t*_: Area under the plasma concentration-time curve from 0 hours to the last measurable concentration
AUMC: Area under the first moment of plasma drug concentration-time curve
*β*: Overall elimination rate constant
BE: Bioavailability enhancer
*C*_max⁡⁡_: Peak concentration
*C*_last_: Last measurable concentration
*C*_ss_: Steady state plasma concentration
Cl_*B*_: Total body clearance
Cl_*F*_: Total plasma clearance
Cl_*R*_: Renal clearance
HPLC: High-performance liquid chromatography
*K*_*a*_: Absorption rate constant
*K*_el_: Elimination rate constant
*K*_*i*_: Ionization constant
*K*_*m*_: Michaelis constant
LD_50_: Half-maximal lethal dose
MIC: Minimum inhibitory concentration
MRT: Mean residence time
RB: Relative bioavailability
*t*_1/2_: Terminal halflife
*t*_1/2*α*_: Absorption half life
*t*_1/2*β*_: Elimination half life
*t*_max⁡_: Time to reach peak concentration
*t*_*d*_: Total duration of pharmacological effect
*V*_*d*_: Volume of distribution
*V*_*d*(area)_: Total area under the plasma drug concentration curve
*V*_*d*(*B*)_: Apparent volume of distribution based on the zero time plasma concentration intercept of the elimination phase
*V*_max⁡_: Maximum enzyme velocity.
